# Discovery of new antimicrobial thiophene derivatives with activity against drug-resistant Gram negative-bacteria

**DOI:** 10.3389/fphar.2024.1412797

**Published:** 2024-08-20

**Authors:** Irene Molina-Panadero, Marcos Morales-Tenorio, Alfonso García-Rubia, Tiziana Ginex, Khalil Eskandari, Ana Martinez, Carmen Gil, Younes Smani

**Affiliations:** ^1^ Andalusian Center of Developmental Biology, CSIC, University of Pablo de Olavide - Seville, Seville, Spain; ^2^ Centro de Investigaciones Biológicas Margarita Salas (CIB-CSIC), Madrid, Spain; ^3^ Department of Molecular Biology and Biochemical Engineering, University of Pablo de Olavide, Seville, Spain

**Keywords:** new thiophene derivatives, antimicrobial resistant drugs, treatment, bacteria, infection, gram-negative

## Abstract

Our aim is to identify new small molecules with antimicrobial potential, especially against colistin-resistant (Col-R) *Acinetobacter baumannii* and *Escherichia coli*. After initial hits identification by fingerprint similarity, MIC of 24 heterocyclic derivatives for *A. baumannii* and *E. coli* reference strains, and bactericidal activity of selected thiophenes against Col-R strains were determined. We analyzed changes in bacterial membrane permeability and the OMPs profile. Additionally, we determined bacterial adherence to host cells and performed molecular docking studies to assess their binding to bacterial targets. The compounds’ MICs ranged from 4 to >64 mg/L. Thiophene derivatives **4**, **5** and **8** exhibited MIC_50_ values between 16 and 32 mg/L for Col-R *A. baumannii* and 8 and 32 mg/L for Col-R *E. coli*. The time-kill curve assay demonstrated that thiophenes **4** and **8** had bactericidal effects against Col-R *A. baumannii* and *E. coli*. Furthermore, treatment with them resulted in increased membrane permeabilization and reduced adherence of these isolates to host cells. Finally, the docking studies showed a stronger binding affinity to CarO1 and Omp33 of *A. baumannii* and OmpW and OmpC of *E. coli*. These findings indicate that thiophene derivatives possess antibacterial activity against Col-R *A. baumannii* and *E. coli*, suggesting that they may enhance the repertoire of drug treatments against bacteria.

## 1 Introduction

Gram-negative bacilli (GNB) are highly efficient in acquiring antimicrobial resistance through genomic, transcriptomic, and proteomic changes (Kung et al., 2010; Yeline et al., 2018). Compounding the problem of antimicrobial resistance is the immediate threat of a reduction in the discovery and development of new antibiotics, as highlighted by the World Health Organization (WHO) (Tacconelli et al., 2018) and other European institutions (O’Neil, 2015; Årdal et al., 2017). Consequently, a perfect storm is converging with regard to these infections: increasing antimicrobial resistance with a decreased development of new drugs (O’Neil, 2015). This context is likely the best example of the purported “Post-Antibiotic Era,” which has relevance even in non-specialized media (Bagley, 2017). It is clear that effective solutions are urgently needed, as stressed by various institutions. Therefore, the development of new strategic antimicrobial therapeutic approaches, such as the use of non-antibiotic substances and/or drug repurposing to be used as monotherapies or in combination with one of the scarce but clinically relevant antibiotics, has become an urgent need.

In this environment, “repurposing” (defined as investigating new uses for existing drugs) has gained renewed interest (Gil et al., 2021a). A variety of drug families have been considered, including anticancer, anthelmintic, anti-inflammatory/immunomodulatory, antipsychotic and antidepressant, statins, and iron-storage drugs (Miró-Canturri et al., 2019; Canturri and Smani, 2023). It has been reported that anticancer drugs like tamoxifen and raloxifene can modify the immune response by regulating cytokine release (Flores et al., 2016; Canturri and Smani, 2023). Previously, we showed *in vitro* and *in vivo* that tamoxifen metabolites have bactericidal activity by reducing bacterial loads *in vitro* and in tissues and fluid of mice (Miró-Canturri et al., 2021a; Miró-Canturri et al., 2021b).

This study was designed to identify new chemical entities with antimicrobial potential. With this aim, we carried out a computational approach combined with experimental confirmation. Initially, a ligand-based virtual screening (LBVS) was performed based on the concept that similar compounds usually show similar biological effects (Maggiora et al., 2014; Muegge et al., 2016). We used two anticancer drugs (4-hydroxytamoxifen and raloxifene) as chemical templates due to their antimicrobial activities previously reported (Ho Sui et al., 2012; Flores et al., 2016; Miró-Canturri et al., 2021c). Using topological fingerprint, we selected 12 chemically diverse compounds from the Medicinal and Biological Chemistry (MBC) library (Ginex et al., 2023) to be experimentally checked as antimicrobials. From the initial set, two thiophene derivatives named AGR1.229 (**1**) and AGR1.230 (**2**), showed interesting activities against reference strains of *Acinetobacter baumannii* and *Escherichia coli*. It is well-documented that many synthetic strategies have allowed the thiophene ring to be present in numerous pharmacologically important compounds (Mabkhot et al., 2016). Various thiophene-based agents have shown antimicrobial activity, such as thiophene-substituted heterocycles (pyridines, azoles, diazines, and azepines, among others), aminothiophenes, compounds based on thiophene carboxylic acids and derivatives, compounds derived from thiophene-2-carboxaldehydes, and metal complexes based on thiophene-containing ligands (for reference, see review by Roman, 2022).

To confirm the results of the two thiophene derivatives AGR1.229 (1) and AGR1.230 (2), we evaluated a focus library of 24 derivatives chemically related to the initial hits, resulting in the identification of thiophenes **4**, **5** and **8** with promising antibacterial activity against *A. baumannii* and *E. coli* resistant to colistin. Preliminar experiments to decipher the mechanism of action of this new class of antibacterials are also presented.

## 2 Materials and methods

### 2.1 Ligand-based virtual screening

**FIGURE 1:**
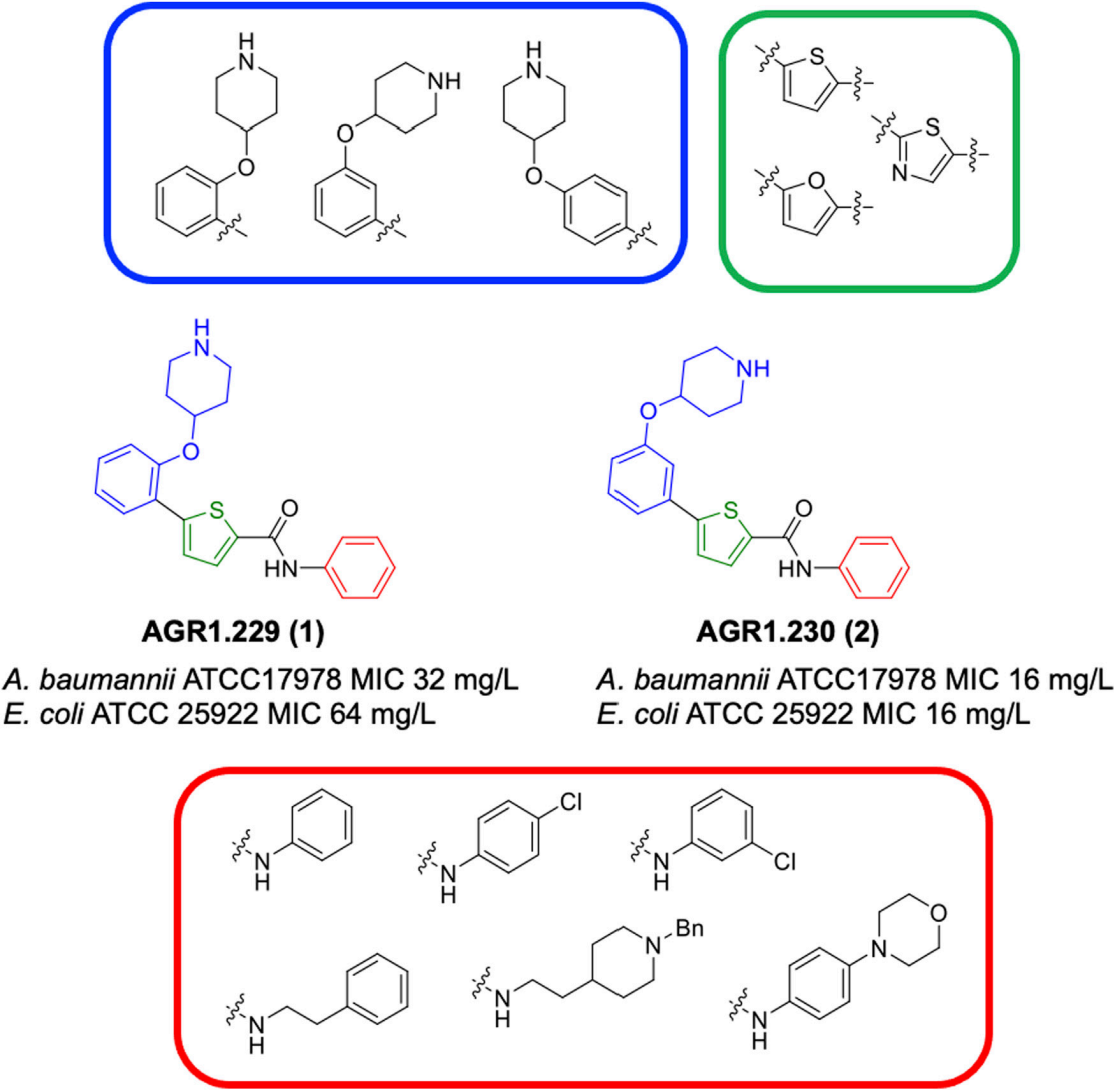
Structural derivatives of thiophenes **1** and **2**.

The MBC library (Ginex et al., 2023) has been *in silico* screened to identify new compounds potentially active against *A. baumannii*, *Pseudomonas aeruginosa* or *E. coli*. The 4 well-known 2D similarity metrics, Buser, Cosine, Kulczynski and Tanimoto have been applied for the prioritization of the potential hits, according to the linear fingerprint method implemented in the similarity-based virtual screening protocol of Schrödinger (Schrödinger Release 2021-1, 2021). The MolPrint2D fingerprints, which encodes atom environment descriptors based on their molecular connectivity table, were used as 2D similarity descriptors (Bender et al., 2004). Two anticancer drugs, 4-hydroxytamoxifen and raloxifene, also reported to have antibacterial effect (Ho Sui et al., 2012; Miró-Canturri et al., 2021b) were selected as reference structures for 2D similarity search (Figure 1). After ranking the compounds of the MBC library based on the 2D similarity metrics mentioned above with tamoxifene and raloxifene, mean values were calculated for each compound considering each metric. Once the compounds were ranked according to mean values, the available ones were selected for evaluation. A total of 12 compounds from similarity-based virtual screening has been selected for further *in vitro* studies. Their similarity indexes with respect to 4-hydroxytamoxifen and raloxifene are reported in Supplementary Table S1 of Supplementary Material.

### 2.2 Compounds studied

All the compounds tested in this work were prepared in the Centro de Investigaciones Biológicas Margarita Salas (CIB-CSIC) following previously described procedures (Corral et al., 1978; Narender et al., 2007; Palomo et al., 2012; Redondo et al., 2012; Gil et al., 2021b; Gil et al., 2023) and are collected in the MBC library (Ginex et al., 2023). Purity has been checked by HPLC (≥95%) and chromatograms are included in Supplementary Figure S1 of Supported Information.

### 2.3 Bacterial strains

A total of eight clinical isolates of *A. baumannii*, 13 clinical isolates of *E. coli* resistant to colistin and two reference strains of *A. baumannii* ATCC 17978 and *E. coli* ATCC 25922 were used in this study. The *A. baumannii* and *E. coli* strains were isolated from the University Hospital of Virgen del Rocío in Seville, Spain and confirmed their species using DNA and MALDI-TOF based confirmation (Valencia et al., 2009; López-Rojas et al., 2011; Gálvez Benítez et al., 2021). The *A. baumannii* ATCC 17978 and *E. coli* MG1655 deficient in Omp33 and OmpC were also used (Smani et al., 2013; Gálvez Benítez et al., 2023).

### 2.4 *In vitro* susceptibility testing

The MICs of thiophene derivatives against reference and colistin-resistant *A. baumannii* and *E. coli*, and MIC of thiophene derivative **4** against *A. baumannii* ATCC 17978 and *E. coli* MG1655 and its isogenic Omp33 and OmpC-deficient strains, respectively, were determined in two independent experiments using the broth microdilution method, in accordance with the standard guidelines of the European Committee on Antimicrobial Susceptibility Testing (EUCAST) (EUCAST, 2022). A 5 × 10^5^ CFU/mL inoculum of each strain was cultured in Luria Bertani (LB) and added to U-bottom microtiter plates (Deltlab, Spain) containing the studied compound. The plates were then incubated for 18 h at 37°C. The MIC_50_, which represent the concentrations that were effective against ≥50% of the isolates tested, were determined.

### 2.5 Time-kill kinetic assays

To determine the bactericidal activity, duplicate time-kill curves were performed for *A. baumannii* Ab11 and Ab21, and *E. coli* MCR1^+^ and R6 MCR1 strains, as previously described (Miró-Canturri et al., 2020). An initial inoculum of 5 × 10^5^ CFU/mL was added to LB in the presence of 1xMIC, 2xMIC and 4xMIC of thiophene derivatives. A drug-free broth was evaluated in parallel as a control. Tubes of each condition were incubated at 37°C with shaking, and viable counts were determined by serial dilution at 0, 2, 4, 8, and 24 h. Viable counts were determined by plating 100 µL of the control, test cultures, or the respective dilutions at the indicated times onto sheep blood agar plates (ThermoFisher, Spain). Plates were incubated for 24 h at 37°C, and after colony counts, the log_10_ of viable cells (CFU/mL) was determined. Bactericidal activity was defined as a reduction of ≥3 log_10_ CFU/mL from the initial inoculum.

### 2.6 Human cell culture

HeLa cells were grown in Dulbecco’s Modified Eagle Medium (DMEM) rich medium containing 10% heat-inactivated fetal bovine serum (FBS), vancomycin (50 mg/L), gentamicin (20 mg/L), and amphotericin B (0.25 mg/L) (Invitrogen, Spain), and 1% HEPES in a humidified incubator with 5% CO_2_ at 37°C. The HeLa cells were routinely passaged every 3 or 4 days. Following this, a 24-well plate was prepared by seeding HeLa cells with a concentration of 1 × 10^5^ cells/mL and incubated it for 24 h in the DMEM rich medium. Immediately before infection, HeLa cells seeded in 24-well plate were washed three times with prewarmed phosphate buffered saline (PBS) and further incubated in DMEM without FBS, vancomycin, gentamicin and amphotericin B (Parra-Millán et al., 2018).

### 2.7 Adhesion assays

HeLa cells were infected with *A. baumannii* Ab11 and Ab21 strains and *E. coli* MCR1^+^ and R6 MCR1 strains at a concentration of 1 × 10^8^ CFU/mL, in the absence and presence of 1xMIC of thiophene derivatives at a multiplicity of infection (MOI) of 100. The bacteria were treated with thiophene derivatives separately for 30 min at 1xMIC before being introduced into the experimental setup. The infection was carried out for 2 h with 5% CO_2_ at 37°C. After that, the infected HeLa cells were washed five times with pre-warmed PBS and lysed with 0.5% Triton X-100. One hundred microliters of the diluted lysates were plated onto LB agar and incubated at 37°C for 24 h to enumerate the developed colonies and determine the number of bacteria that had attached to the HeLa cells (Parra-Millán et al., 2018).

### 2.8 Membrane permeability assays

The bacterial cells were grown in LB broth and incubated in the absence or presence of 0.5xMIC of thiophene derivatives for 3 h. The pellet was harvested by ultracentrifugation at 4600*g* for 15 min. The bacterial cells were washed with PBS 1x, and after centrifugation in the same condition described before, the pellet was resuspended in 100 µL of PBS 1x containing 10 μL of Ethidium Homodimer-1 (EthD-1) (Invitrogen, United States). After 10 min of incubation, (i) 10 μL of the pellet was placed on a slide to visualize it using a Zeiss fluorescence microscope (Zeiss MicroImaging GmbH, Germany), and (ii) 100 μL was placed into a 96-well plate to measure fluorescence for 300 min using a Typhoon FLA 9000 laser scanner (GE Healthcare Life Sciences, United States) and quantified using ImageQuant TL software (GE Healthcare Life Sciences, United States) (Miró-Canturri et al., 2020).

### 2.9 Analysis of outer membrane proteins

To assess the effect of thiophene derivatives on outer membrane proteins (OMPs), bacterial cells of colistin-resistant *A. baumannii* Ab11 and Ab21 strains, and colistin-resistant *E. coli* MCR1^+^ and R6 MCR1 strains were grown in LB broth to logarithmic phase, followed by treatment with 0.5xMIC of the compounds for 24 h. The cells were then lysed by sonication, and OMPs were extracted with sodium lauryl sarcosinate (Sigma, Spain) and recovered by ultracentrifugation, as described previously (Miró-Canturri et al., 2021b). The extracted OMPs were separated by sodium dodecyl sulfate polyacrylamide gel electrophoresis (SDS-PAGE) using 10% SDS gels, and 6 μg of OMP protein was loaded per lane. The gels were stained with SimplyBlue SafeStain (Invitrogen, Spain) to visualize the OMP profiles.

### 2.10 Docking and molecular modelling

All chemical structures were drawn and optimized by ChemOffice Suite released in 2022 (Rakshit et al., 2023). All molecular docking calculations and preparation of protein and ligand systems were done with the Schrödinger software package (Friesner et al., 2006; Gentile et al., 2022). Protein structures of the transmembrane domain of *A. baumannii* OmpA and OmpW were obtained by homology modeling using I-TASSER online server (http://zhanglab.ccmb.med.umich.edu/I-TASSER/) (Yang et al., 2015). The amino acid sequences of OmpA and OmpW of *A. baumannii* were taken from Genbank (ID: AXV53527.1 and SUU48067.1, respectively), and OmpA and OmpW crystal structures of *E. coli* (PDB IDs: 1bxw and 2f1v, respectively) were applied as template (Hong et al., 2006; Vila-Farrés et al., 2017). The percentage of identity between OmpA of *E. coli* and *A. baumannii*, and OmpW of *E. coli* and *A. baumannii* obtained by I-TASSER was 26% and 32%, respectively.

The crystal structures for the rest of the OMPs were taken from the Protein Data Bank (PDB) (PDB ID: OMPs of *A. baumannii*; CarO1: 4rl9, CarO2: 4rlb, Omp33: 6gie, and OMPs of *E. coli*; OmpC: 2j1n, OmpF: 4gcq) (Baslé et al., 2006; Ziervogel et al., 2013; Zahn et al., 2015; Abellón-Ruiz et al., 2018). All PDB entries (4rl9, 4rlb, 6gie, 2j1n, 4gcq) obtained from X-Ray crystals were used in all docking studies. All models were prepared with Protein Preparation Wizard, minimized, and a post-docking minimization was performed to further improve the accuracy of the results (Schrödinger Release 2013-3, 2013). The SiteMap module of the Schrödinger Suite identified a potentially druggable area for ligand binding in the border of the opening of the transmembrane domain of OMPs and flexible extracellular loops (Schrödinger Release 2013-3, 2013). The same area was used for constructing docking grids for Glide. The chemical structure of selected thiophene derivative **4** was built, and optimized by LigPrep (Schrödinger Release 2013-3, 2013), and additional MacroModel conformational search techniques were applied to this compound. The best and most stable pose was submitted for ligand-protein docking simulations. The ligand was docked using Glide XP extra precision and furthermore, the OPLS4 force field through sampling flexible ligand structures as well as ring conformations and nitrogen inversions were applied.

### 2.11 Statistical analysis

Group data are presented as means ± standard errors of the means (SEM). The Student’s t-test was used to determine differences between means using the GraphPad Prism 9. A *p*-value < 0.05 was considered significant.

## 3 Results

### 3.1 Ligand-based virtual screening using topological indexes

Due to their reported antibacterial properties (Ho Sui et al., 2012; Miró-Canturri et al., 2021b), 4-hydroxytamoxifen and raloxifene were selected as reference structures for 2D similarity search to identify new antibacterial compounds. With this aim, we selected the MBC library to be screened *in silico*. MBC library is a suitable reservoir of hits for drug discovery due to its high quality in terms of structural diversity and drug-like properties. Moreover, physicochemical and pharmaceutical properties have been calculated and compared with those of other well-known publicly available libraries corroborating this fact (Ginex et al., 2023). For the prioritization of compounds, four well-known 2D similarity metrics, Buser, Cosine, Kulczynski and Tanimoto indexes were used (Bender et al., 2004). A total of 12 heterocyclic compounds from LBVS were selected for further *in vitro* studies (Supplementary Table S1). Their similarity indexes with respect to 4-hydroxytamoxifen and raloxifene are reported in Supplementary Table S2 of Supporting Information.

### 3.2 *In vitro* activity of thiophene derivatives in *Acinetobacter baumannii* and *Escherichia coli* reference strains

Compounds initially selected were screened using a microdilution assay against *A. baumannii* ATCC 17978 and *E. coli* ATCC 25922 strains to determine the MIC. Two of them, derivatives AGR1.229 (**1**) and AGR1.230 (**2**) showed MICs ranging from 16 to 64 mg/L (Supplementary Table S1). Interestingly, both have a central thiophene scaffold disubstituted with a benzamide group and a phenyl ring attached at positions 2 and 5 respectively. A piperidine linked by an oxygen atom in *ortho* and *meta* position respectively is also present in the molecules. These interesting activities motivated us to evaluate different structurally related thiophenes in the same initial screening (Figure 1). Such is the case of 12 new structurally related thiophenes, compounds **4**-**15**, with different substituents into the amide group linked to the thiophene ring and with the piperidin-4-yloxy group in *ortho*, *meta* and *para* position. Additionally, 12 more compounds where the heterocyclic central scaffold was changed to furan or thiazole moieties were also evaluated, compounds **16**-**23** and **24**-**27** respectively, in order to see the influence of the main heterocycle in the antibacterial activity. In total 24 new compounds can be found in Table 1 together with their antimicrobial activities. From them, 14 derivatives exhibited MICs ranging from 4 to 64 mg/L (Table 1).

**TABLE 1:** Antibacterial activity of new heterocyclic compounds in *Acinetobacter baumannii* and *Escherichia coli* reference strains.

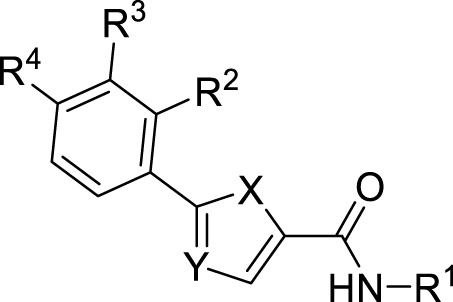								
	R^1^	R^2^	R^3^	R^4^	Y	X	*A.baumannii* ATCC 17978 MIC (mg/L)	*E.coli* ATCC 25922 MIC (mg/L)
**AGR1.229 (1)**	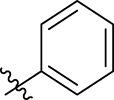	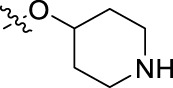	H	H	CH	S	**32**	**64**
**AGR1.230 (2)**	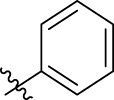	H	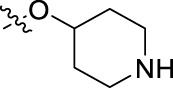	H	CH	S	**16**	**16**
**AGR1.231 (3)**	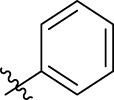	H	H	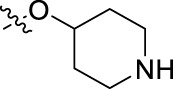	CH	S	>64	>64
**4**	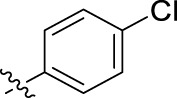	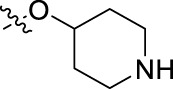	H	H	CH	S	**4**	**8**
**5**	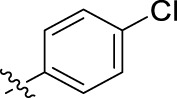	H	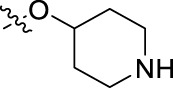	H	CH	S	**16**	**16**
**6**	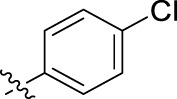	H	H	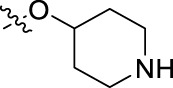	CH	S	>64	>64
**7**	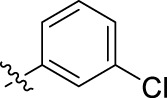	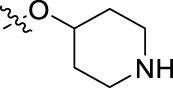	H	H	CH	S	>64	**8**
**8**	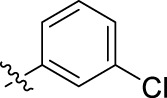	H	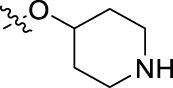	H	CH	S	**64**	**32**
**9**	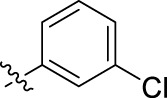	H	H	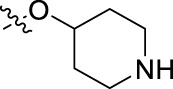	CH	S	**64**	**32**
**10**	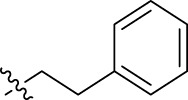	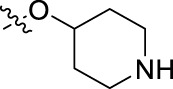	H	H	CH	S	**32**	**64**
**11**	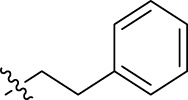	H	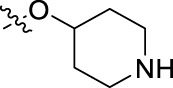	H	CH	S	>64	>64
**12**	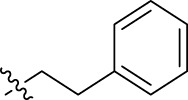	H	H	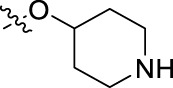	CH	S	>64	>64
**13**	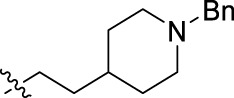	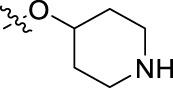	H	H	CH	S	**64**	**64**
**14**	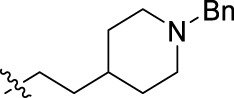	H	H	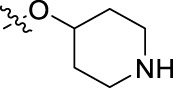	CH	S	>64	>64
**15**	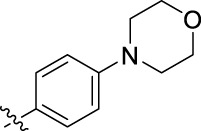	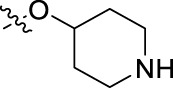	H	H	CH	S	>64	>64
**16**	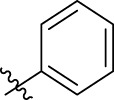	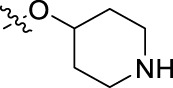	H	H	CH	O	**32**	**32/64**
**17**	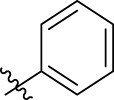	H	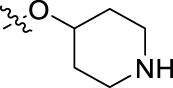	H	CH	O	>64	>64
**18**	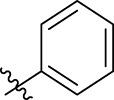	H	H	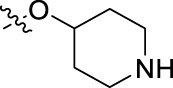	CH	O	**64**	>64
**19**	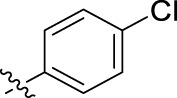	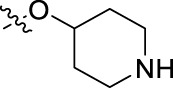	H	H	CH	O	**8**	**16**
**20**	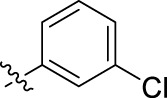	H	H	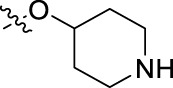	CH	O	**16**	**32**
**21**	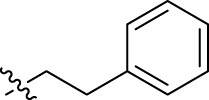	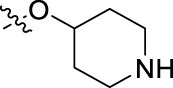	H	H	CH	O	**64**	>64
**22**	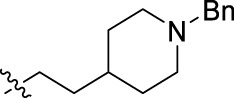	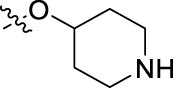	H	H	CH	O	>64	>64
**23**	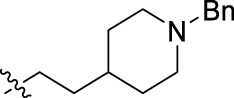	H	H	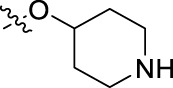	CH	O	>64	>64
**24**	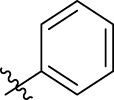	H	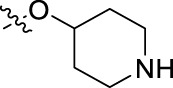	H	N	S	>64	>64
**25**	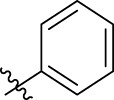	H	H	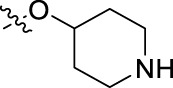	N	S	>64	>64
**26**	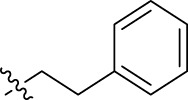	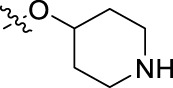	H	H	N	S	32/>64	32/>64
**27**	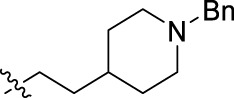	H	H	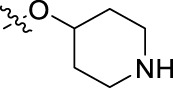	N	S	>64	>64

MIC: minimal inhibitory concentration. Bold values represent MIC values < = 64

Regarding initial evaluated thiophene derivatives (AGR1.229 (**1**), AGR1.230 (**2**) and AGR1.231 (**3**)), the three of them have a benzamide in position 2 and piperidin-4-yloxy group in *ortho*, *meta* and *para* respectively. When this substituent is in *ortho* position (thiophene **1**), the MIC in *A. baumannii* and *E. coli* was reduced with respect to the MIC of thiophene **3** with the substituent in *para* to 32 and 64 mg/L. A more potent reduction was observed when the substituent was in *meta* (thiophene **2**) reaching a MIC level of 16 mg/L against both pathogens.

Substitution in the amide of position 2 of the thiophene ring with a 4-chlorophenyl substituent led to a more potent compound in the case of *o*-derivatives (**4** vs*.* **1**), but in the case of *m*-derivatives (**5** vs*.* **2**) did not improve the MIC, which remained at 16 mg/L against both pathogens. Finally, *p*-derivatives (**6** vs*.* **3**) remained inactive.

When thiophenes were substituted with a 3-chlorophenyl moiety the *o*-derivative **7** showed a MIC in *E. coli* of 8 mg/L but a complete lack of activity in *A. baumannii* whilst the *m*-derivative **8** did not show an improved MIC. Lastly, *p*-derivative **9** showed a reduced MIC in both pathogens with respect to inactive analogs **3** and **6**.

Furthermore, the change of the phenyl substituent of the amide by phenetyl substituent led to inactive compounds in the case of *m*- and *p*-derivatives **11** and **12**, respectively, while the *o*-derivative **10** did not change the MIC with respect to the parent compound (thiophene **1**), which remained at 32 and 64 mg/L against *A. baumannii* and *E. coli*, respectively.

Introduction of substituents in the amide like 2-(1-benzylpiperidin-4-yl)ethyl or morpholinophenyl (thiophenes **13**, **14** and **15**) did not seem to be critical for potentiating the antibacterial activity, as the MIC values were >64 mg/L in all the cases.

Moreover, when thiophene ring was substituted by furan and thiazole core no significant improvement was observed with respect to the parent thiophene hit (e.g., furan **16** vs*.* thiophene **1**, furan **19** vs*.* thiophene **4** or furan **20** vs*.* thiophene **9**).

As a next step, those compounds with a MIC ≤64 mg/L for both pathogens were evaluated against 16 clinical strains of Col-R *A. baumannii* and *E. coli* to extend their antibacterial effect to antimicrobial resistant strains. Of note, the MIC_50_ values for colistin are 128 mg/L and 8 mg/L for Col-R *A. baumannii* and *E. coli*, respectively. As shown in Table 2, compounds **4**, **5** and **8** exhibited stronger antibacterial activity against Col-R *A. baumannii*, with an MIC_50_ (minimum concentrations that inhibit 50% of the screened strains) value of 16, 16 and 32 mg/L, respectively, and against Col-R *E. coli*, with MIC_50_ values of 8, 32 and 32 mg/L, respectively. However, compounds **1**, **2**, **9**, **10**, **13**, **16**, **19** and **20** showed higher MIC_50_ values ranging from 64 to >64 mg/L to both pathogens.

**TABLE 2:** MIC of selected thiophene derivatives and colistin for clinical colistin-resistant *Acinetobacter baumannii* and *Escherichia coli*.

MIC (mg/L)													
Pathogen	Strain	Colistin	1	2	4	5	8	9	10	13	16	19	20
*A. baumannii*	RC-64	32	64	16	8	16	32	>64	64	>64	64	32	>64
	Ab11	256	64	32	16	4	16	8	>64	>64	64	32	>64
	Ab20	64	64	32	16	16	32	>64	>64	>64	64	32	>64
	Ab21	128	16	32	4	>64	64	>64	16	>64	64	16	>64
	Ab22	128	16	8	4	16	32	>64	32	>64	64	8	32
	Ab99	64	64	32	8	64	64	>64	>64	>64	64	16	64
	Ab113	256	64	32	16	>64	32	>64	>64	>64	64	16	64
	MIC_50_	128	64	**32**	**16**	**16**	**32**	>64	>64	>64	64	**16**	>64
*E. coli*	CRA5	8	64	32	4	32	32	>64	64	>64	>64	16	64
	CRA7	8	64	>64	8	>64	64	>64	64	>64	64	32	>64
	CRA8	8	64	>64	16	>64	64	>64	64	>64	64	64	>64
	CRA17	8	64	64	8	32	32	>64	64	>64	64	16	64
	CRA20	8	64	>64	16	>64	64	>64	64	>64	>64	>64	>64
	CRA32	8	64	64	4	32	16	64	64	>64	64	16	64
	CRA57	4	64	32	4	32	16	64	64	>64	>64	8	8
	MCR1^+^	4	>64	>64	16	32	>64	>64	64	>64	>64	>64	>64
	R6 MCR1	8	ND	ND	16	16	16	>64	>64	>64	64	>64	32
	MIC_50_	8	64	>64	**8**	**32**	**32**	>64	64	>64	64	64	64

MIC, minimal inhibitory concentration. Bold values represent MIC50 < = 32

### 3.3 Time-kill curves

**FIGURE 2:**
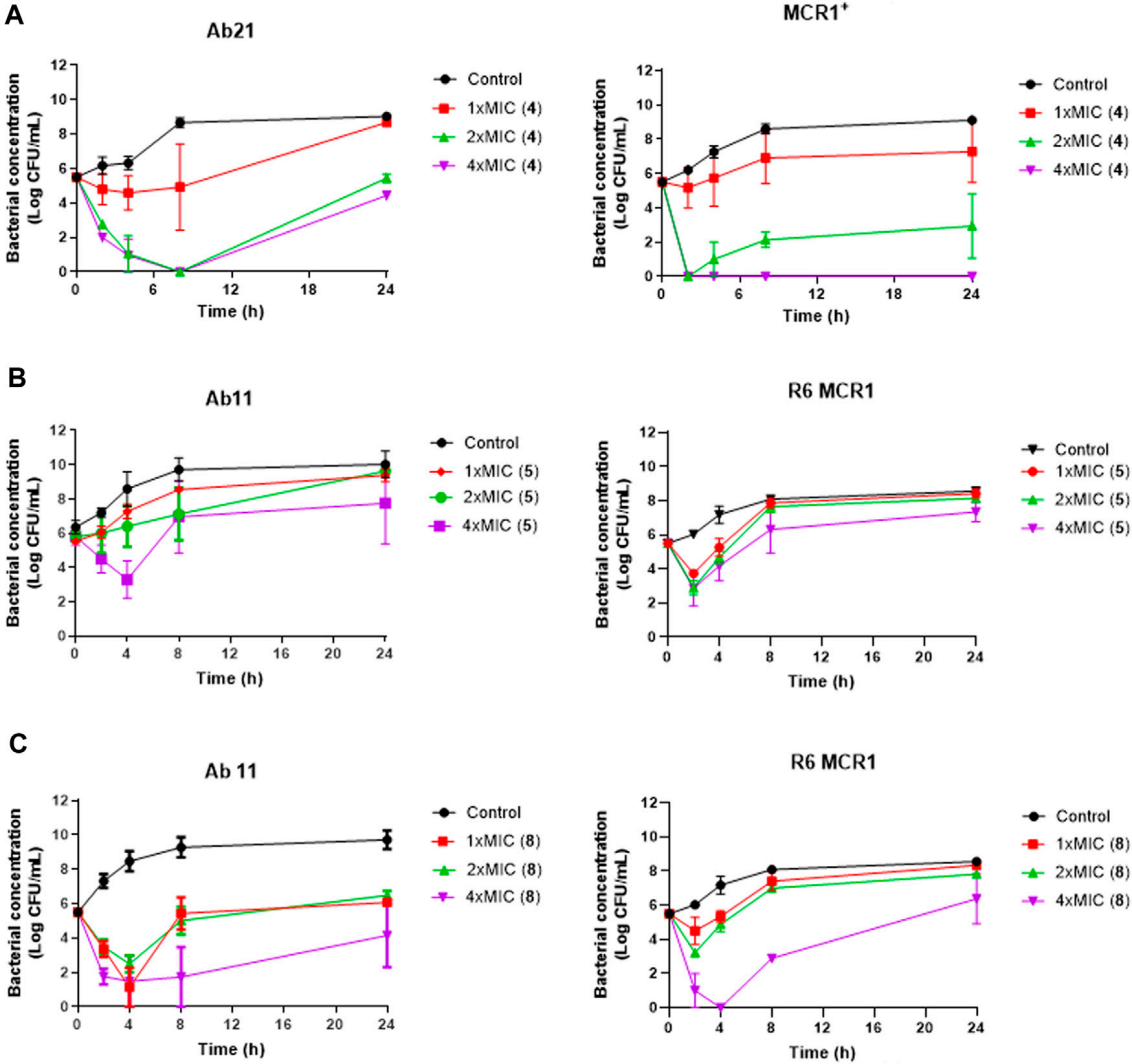
Antibacterial activity of thiophene derivatives 4, 5 and 8 at different concentrations against clinical colistin-resistant *A. baumannii* and *E. coli*. **(A)** Time-kill curves of *Acinetobacter baumannii* Ab21 and *Escherichia coli* MCR1^+^ strains in the presence of 1x and 1x, 2x and 4xMIC thiophene **4** for 24 h. The thiophene **4** MIC for Ab21 and MCR1^+^ strains is 4 and 16 mg/L. **(B, C)** Time-kill curves of *Acinetobacter baumannii* Ab11 and *Escherichia coli* R6 MCR1 strains in the presence of 1x and 1x, 2x and 4xMIC thiophene **5** and **8**, respectively, for 24 h. The thiophene **5** MIC for Ab11 and R6 MCR1 strains is 4 and 16 mg/L, and thiophene **8** MIC for Ab11 and R6 MCR1 strains is 16 mg/L, and Data are represented as mean from two independent experiments.

Using time-course assays, we evaluated the bactericidal and bacteriostatic activities of the most potent thiophene derivatives (**4**, **5** and **8**, Figure 1) against *A. baumannii* Ab11 and Ab21 strains, and *E. coli* MCR1^+^ and R6 MCR1 strains. Figure 2 illustrates that thiophene **4** at 2xMIC and 4xMIC exhibited bactericidal effects after 8 h treatment, reducing the bacterial count of the Ab21 strain by over 5.5 log_10_ CFU/mL. For the MCR1^+^ strain, thiophene **4** at 2xMIC and 4xMIC presented bactericidal activity after 24 h, reducing the bacterial count by over 3 and 5.5 log_10_ CFU/mL, respectively.

In the case of thiophene **5**, no bactericidal activity against Ab11 and R6 MCR1 strains was found at 1xMIC, 2xMIC, and 4xMIC during 24 h (Figure 2).

For thiophene **8**, bactericidal activity against Ab11 and R6 MCR1 strains was found at 4xMIC at 8 h post-treatment. In contrast, this compound at 1x and 2xMIC did not show bactericidal activity during 24 h (Figure 2). Of note, and an exception for thiophene **4** at 4xMIC against MCR1^+^ strain, the complete inhibition of bacterial growth was not achieved by any of the compounds.

### 3.4 Effect of thiophene derivatives on the bacterial cell membrane

**FIGURE 3:**
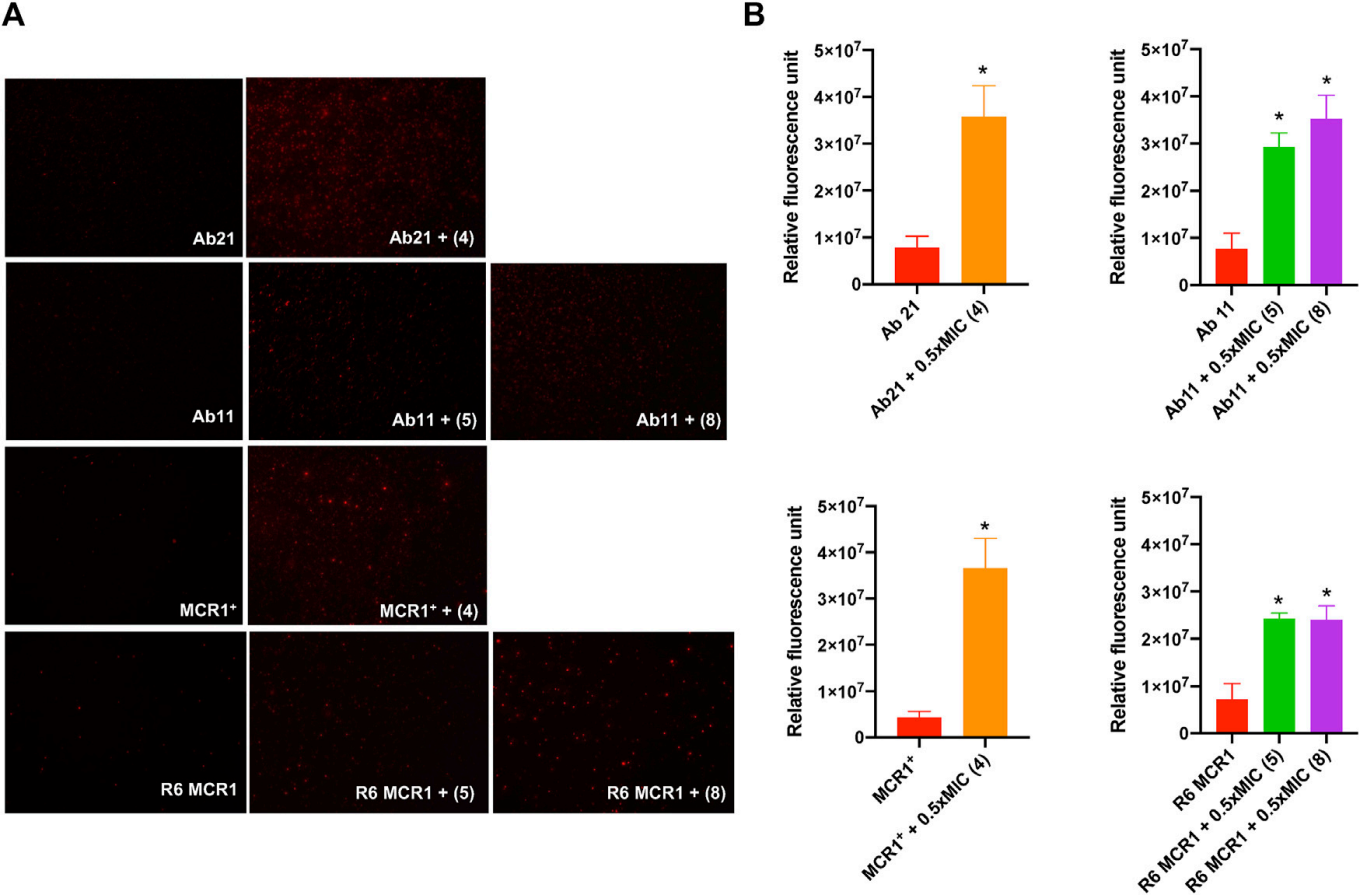
Effect of thiophene derivatives 4, 5 and 8 on the bacterial permeability against clinical colistin-resistant *A. baumannii* and (E) *coli*. **(A)** The membrane permeabilization of colistin-resistant *Acinetobacter baumannii* and *Escherichia coli* strains in the absence and presence of 0.5xMIC of thiophenes **4**, **5** and **8**, using fluorescence microscopy. **(B)** The membrane permeabilization of colistin-resistant *Acinetobacter baumannii* and *Escherichia coli* strains in the absence and presence of 0.5xMIC of thiophenes **4**, **5** and **8**, incubated for 10 min, was quantified by Typhon Scanner. Data are represented as mean ± SEM from three independent experiments. *Acinetobacter baumannii* Ab21 and *Escherichia coli* MCR1^+^ strains were treated with thiophene **4**. *Acinetobacter baumannii* Ab11 and *Escherichia coli* R6 MCR1 strains were treated with thiophenes **5** and **8**. The thiophene **4** MIC for Ab21 and MCR1^+^ strains is 4 and 16 mg/L, the thiophene **5** MIC for Ab11 and R6 MCR1 strains is 4 and 16 mg/L, and the thiophene **8** MIC for Ab11 and R6 MCR1 strains is 16 mg/L *p < 0.05 untreated cells compared with thiophene derivative treatment.

In order to determine the mode of action of the thiophenes **4**, **5** and **8**, we examined their effect on the membrane permeability of *A. baumannii* Ab11 and Ab21 strains, and *E. coli* MCR1^+^ and R6 MCR1 strains, by incubating them with ethidium homodimer-1, a DNA stain that is unable to cross intact membranes. Fluorescence microscopy analysis showed an increase in cellular fluorescence of the Ab21 and MCR1^+^ strains in the presence of 0.5xMIC of compound **4**, and the Ab11 and R6 MCR1 strains in the presence of 0.5xMIC of compounds **5** or **8**, with a higher effect observed in the case of treatment with compound **4** (Figure 3A).

Moreover, fluorescence monitoring using a Typhoon FLA scanner for 3 h confirmed the results obtained by fluorescence microscopy, indicating that the three thiophene derivatives significantly increase the membrane permeability of these strains (Figure 3B). Basal levels of fluorescence were observed in the control. These data suggest that thiophene **4**, **5** and **8** affect the integrity of the bacterial cell wall of *A. baumannii* and *E. coli*.

### 3.5 Effect of thiophene derivatives on the OMP profiles

The OMP profiles of the *A. baumannii* strains Ab11 and Ab21, and *E. coli* strains MCR1^+^ and R6 MCR1, did not show significant changes between the control and treatment conditions after incubation for 24 h with 0.5xMIC of thiophenes **4**, **5** and **8** (Supplementary Figure S2). These results suggest that the thiophene derivatives did not affect the expression of OMPs.

### 3.6 Effect of thiophene derivatives on the bacterial adherence to host cells

**FIGURE 4:**
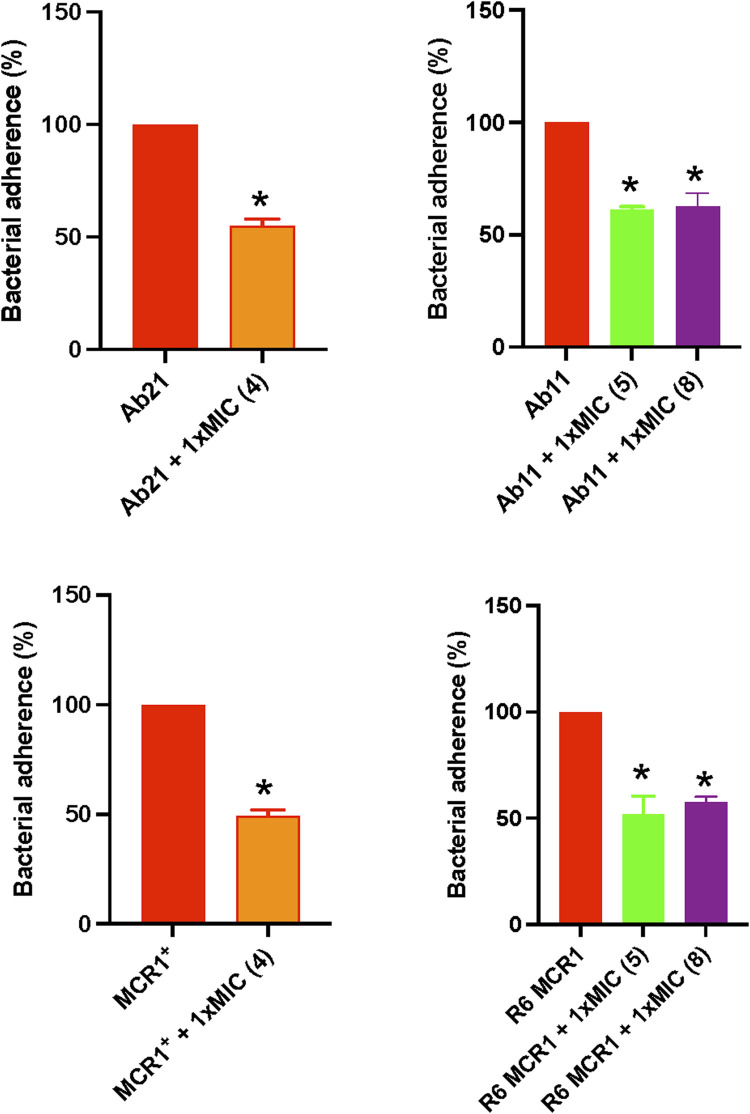
Effect of thiophene derivatives 4, 5 and 8 on clinical colistin-resistant *Acinetobacter baumannii* and *Escherichia coli* interaction with host cells. Bacteria were treated with thiophene **4** (1xMIC)**, 5** (1xMIC) and **8** (1xMIC) for 30 min before to infect HeLa cells. The assay of *Acinetobacter baumannii* and *Escherichia coli* adherence to HeLa cells for 2 h was performed as described under “Materials and Methods section”. The thiophene **4** MIC for Ab21 and MCR1^+^ strains is 4 and 16 mg/L, the thiophene **5** MIC for Ab11 and R6 MCR1 strains is 4 and 16 mg/L, and the thiophene **8** MIC for Ab11 and R6 MCR1 strains is 16 mg/L. Data are represented as mean ± SEM from three independent experiments. *, *p* < 0.05 untreated cells compared with thiophene derivative treatment.

To evaluate the effect of selected thiophenes on *A. baumannii* and *E. coli* interaction with host cells, we studied the adherence of Ab11, Ab21, MCR1^+^, and R6 strains to HeLa cells for 2 h in the presence of the compounds. Treatment with thiophene **4** at 1xMIC reduced the adherence of Ab21 and MCR1^+^ strains to HeLa cells by 47% (*p* < 0.05) and 51% (*p* < 0.05), respectively. Notably, a lesser significant reduction was observed with thiophenes **5** or **8**, where both compounds reduced the adherence of Ab11 strain by 38% (*p* < 0.05) and 37% (*p* < 0.05), respectively, and the adherence of R6 MCR1 strain by 48% (*p* < 0.05) and 42% (*p* < 0.05), respectively (Figure 4).

### 3.7 Docking and molecular modelling

As thiophene **4** has shown better antimicrobial resistant activity in terms of MIC_50_, time-kill curve, cell permeability, and bacterial adherence to host, we next selected this compound for the following studies. To throw some light inside its mechanism of action, compound **4** was subsequently docked in the transmembrane (TM) domain of different outer membrane proteins such as OmpA, Omp33, OmpW, and CarO1 of *A. baumannii* and ranked on the basis of the scoring function (see “Methods” section). As no three-dimensional structures of the TM domain of *A. baumannii* OmpA and OmpW were found in the PDB, a homology model using the I-TASSER server was built using the amino acid sequence for OmpA and OmpW of *A. baumannii* (GenBank: AXV53527.1, and ADX03096.1 respectively) using as templates the known 3D structures of OmpA and OmpW of *E. coli* (PDB IDs: 1qjp, and 2f1v respectively). Similar docking studies were performed for the OmpA, OmpW, OmpC, and OmpF of *E. coli*.

**FIGURE 5:**
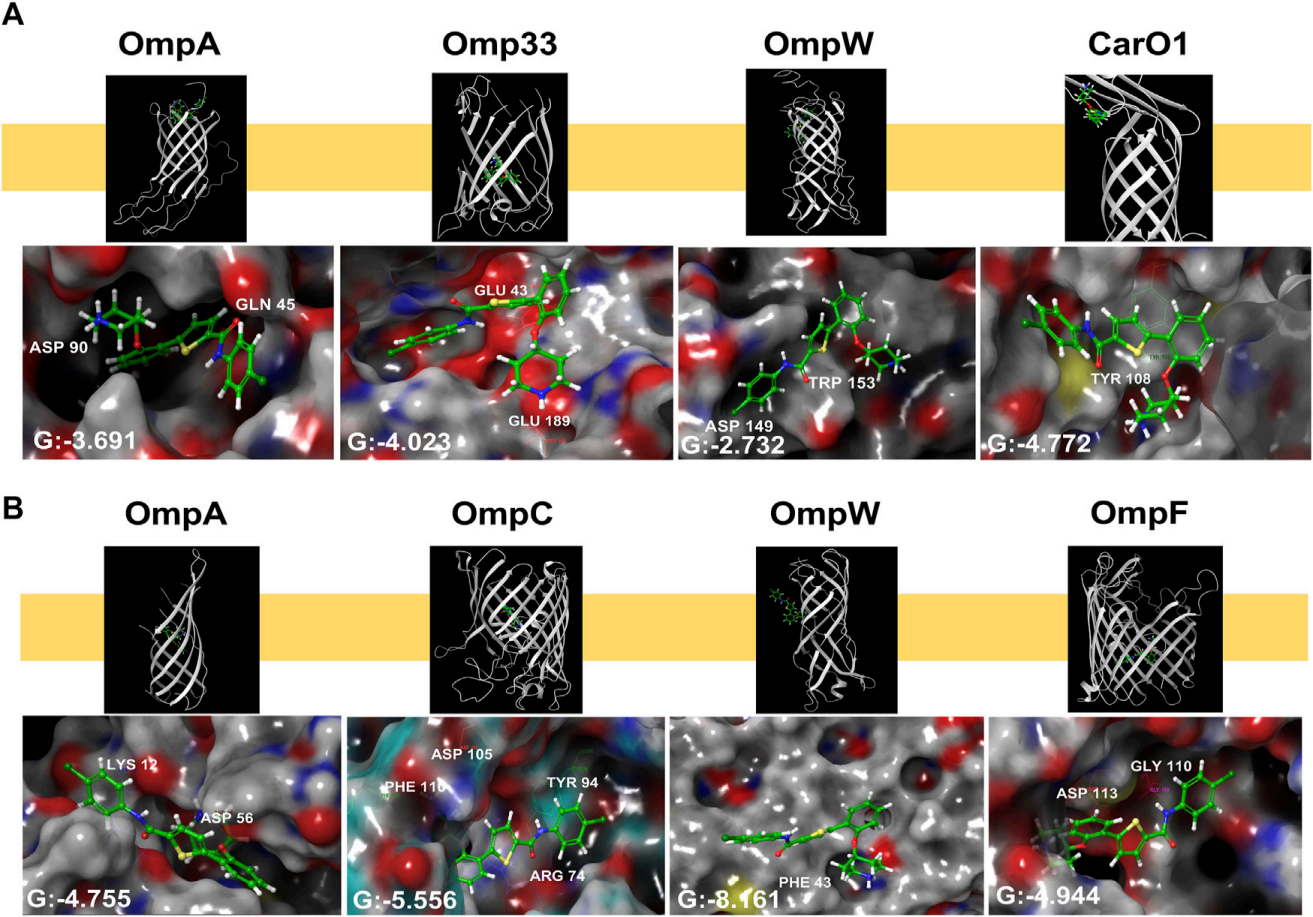
Structural models generated by docking of thiophene derivative **4** into the transmembrane domain of *Acinetobacter baumannii* Omp33 and CarO1 **(A)** and *Escherichia coli* OmpA, OmpC, OmpW and OmpF **(B)**, and into the homology model of *Acinetobacter baumannii* OmpA and OmpW generated as described under “Methods”. Thiophene **4** is displayed as sticks. G: Glide score.

Thiophene **4** showed better docking score in *A. baumannii* CarO1and Omp33 and *E. coli* OmpW and OmpC compared to two other porins (Supplementary Table S3). The most stable pose shows that thiophene **4** binds to Tyr108 of CarO1, and has a powerful interactions with Phe43 of OmpW through π-π stacking bonds with the thiophene ring (Figure 5). The activity of thiophene **4** against *A. baumannii* ATCC 17978 and *E. coli* MG1655 is lower when both strains are deficient in Omp33 and OmpC, by increasing MIC 2 and 4 times, respectively (Table 3).

**TABLE 3:** MIC of thiophene derivative **4** for *Acinetobacter baumannii* ATCC 17978 and *Escherichia coli* MG1655 and its isogenic Omp33 and OmpC-deficient strains, respectively.

	Thiophene 4	Fold change
ATCC 17978 wt	4	-
ATCC 17978 ΔOmp33	8	**2**
MG1655	16	-
MG1655ΔOmpC	64	**4**

Bold value represent fold change > = 2 times.

## 4 Discussion

The emergence of multidrug-resistant GNB (MDR-GNB) has prompted the use of colistin as a last resort in the treatment of severe infections caused by these pathogens. Although uncommon, colistin resistance is increasing, and its spread is considered a global health threat (Giamarellou, 2016).

Due to the antibacterial effect of tamoxifen and its metabolites in *A. baumannii* and *E. coli* (Miró-Canturri et al., 2021b; Miró-Canturri et al., 2021c), we hypothesized that thiophene derivatives identified in this study after a similarity search based on fingerprint analysis, may exhibit good antibacterial activity against *A. baumannii* and *E. coli* resistant to colistin. From initial experimental confirmation of thiophenes AGR1.229 (**1**) and AGR1.230 (**2**), a total of new 24 compounds including thiophene and related furan and thiazole derivatives were initially screened against *A. baumannii* ATCC 17978 and *E. coli* ATCC 25922 strains (Table 1). Some of these derivatives showed MICs ranging from 4 to 64 mg/L. It is noteworthy that thiophene derivatives did not show antibacterial activity against *P. aeruginosa* (data not shown), consistent with previously published data on the antibacterial activity of the anticancer drug family tamoxifen and tamoxifen metabolites used as starting points of this work (Miró-Canturri et al., 2021b; Miró-Canturri et al., 2021c).

The MIC_50_ values for thiophenes **4**, **5** and **8** against Col-R *A. baumannii* were 16, 16 and 32 mg/L, respectively, while for Col-R *E. coli*, were 8, 32, and 32 mg/L, respectively (Table 2), which overall fell within the range of other known antibiotics such as amikacin, amoxicillin-clavulanic, ceftazidime-avibactam, fosfomycin, among others (EUCAST, 2024). However, the MIC_50_ values for the other thiophene derivatives were higher than 32 mg/L. Reasons for this difference could be related to the chemical structure differences between these derivatives. The three thiophene derivatives selected for further studies have a substituent with chlorine (Figure 1).

The antibacterial activity of the thiophene **4** at 2x and 4xMIC against Col-R strains of *A. baumannii* and *E. coli* is higher than that of thiophenes **5** and **8** (Figure 2). This result may be related to the position of the piperidin-4-yloxy group in the structure. While in thiophene **4**, it is located in *ortho* position of the phenyl group attached to the 5-position of the thiophene, in derivatives **5** and **8**, is attached in *para*.

Moreover, the piperidin-4-yloxy group of the thiophenes **5** and **8** is located in the same position *para*; however, the thiophene **8** at 4xMIC showed more antibacterial activity in the first 6 h of bacterial growth than thiophene **5**. This could be related to the chlorophenyl position in the R1.

The antimicrobial activity of the selected derivatives identified in this work hints at a promising potential that deserves to be further explored *in vivo* after determining their pharmacokinetic parameters. However, *in vitro* bacterial growth showed a progressive regrowth of the Col-R *A. baumannii* and Col-R *E. coli* strains after treatment with thiophene **4** at 2xMIC and 4xMIC, suggesting that the *A. baumannii* and *E. coli* strains have acquired resistance to this derivative. Nevertheless, it should be noted that the MICs of thiophene **4** against for Ab21 and MCR1^+^ strains in this time-kill assays condition were 64 and 32 mg/L, respectively, which are below the 4x MIC of the thiophene **4**. Further investigations, including the determination of its concentration during the time-kill assay, are necessary to better understand the regrowth of these strains in the presence of this compound.

Additionally, OMP profiles of *A. baumannii* and *E. coli* strains were not modified after incubation with the three selected thiophene derivatives, indicating that changes in OMP expression could not be related to the mechanism of action of these compounds. Miro Canturri et al. reported similar findings, where tamoxifen metabolites did not alter the expression of OMPs of *A. baumannii* and *E. coli* MDR strains (Miró-Canturri et al., 2021b). However, we demonstrated that these thiophene derivatives increased the membrane permeability of *A. baumannii* and *E. coli* strains (Figure 3). Similar results were observed in studies on tamoxifen metabolites (Miró-Canturri et al., 2021a; Miró-Canturri et al., 2021b). It is known that the mechanism of action of the anticancer drug tamoxifen in fungi is related to its binding to calmodulin (Dolan et al., 2009; Butts et al., 2015). Additionally, Scott et al. showed that 4-hydroxytamoxifen, a tamoxifen metabolite, might inhibit bacterial phospholipase D (Scott et al., 2015). Further studies on the mechanism of action of thiophene derivatives against *A. baumannii* and *E. coli* and their therapeutic efficacy in animal experimental models of infection would be of interest.

It is widely known that OMPs are among the most relevant bacterial targets for antibacterial compounds (Rosas and Lithgow, 2021). Tamoxifen and its metabolites, used here as a chemical template to search similar compound based on topological indexes, are widely associated with the inhibition of bacterial cell membrane function (Luxo et al., 2003; El Arbi et al., 2014; Feng et al., 2015). This is primarily due to the hydrophobic nature of tamoxifen, stemming from the presence of alkyl groups attached to the amino group in its structure (Levinson, 2017). Additionally, a recent study from our group showed that 4-hydroxytamoxifen, a major tamoxifen metabolite, binds rigorously to OmpW of *A. baumannii* (Vila Dominguez et al., 2022).

For these reasons, we decided to carry out a preliminary computational study to determine if thiophene **4** potentially bind to OMPs. Eight porins were considered, four from *A. baumannii* (OmpA, Omp33, OmpW and CarO1) and four from *E. coli* (OmpA, OmpC, OmpW and OmpF). As a result, this compound exhibited a better docking score in CarO1 and Omp33 of *A. baumannii* and OmpW and OmpC of *E. coli* compared to other porins of *A. baumannii* and *E. coli*. The results revealed a potent π-π stacking interaction between Tyr108 of CarO1 and Phe43 of OmpW and the thiophene ring. For Omp33, thiophene **4** has an affinity for the structure of glutamic acids (Glu189 and Glu43) through its piperidinyl ring. However, for OmpC, this compound shows a strong tendency to bind to Arg74, Phe110, Asp105, and Tyr94 through its carbonyl group, piperidinyl ring, piperidinyl ring, and carbonyl group, respectively. These interactions are so potent in a way that they lead to much greater stability of ligand **4** in the binding pocket to these OMPs. Omp33 and OmpC constitute the main OMPs in *A. baumannii* and *E. coli* that are necessary for drug transport across cellular membranes (Smani et al., 2013; Rumbo et al., 2014; Bafna et al., 2020). Biological confirmatory assays showed that their deficiencies in *Acinetobacter bauamnnii* and *E. coli*, respectively, allowed both pathogens to be less susceptible to thiophene **4**.

The findings of this study showed that the existence of a thiophene ring, an amide group, piperidinyl and carbonyl groups plays a crucial role in the chemical structure of the ligand to interact with OMPs and subsequently to show antimicrobial activity. Thus, it is plausible that thiophene **4** have more than one OMP target in *A. baumannii* which may explain the higher antibacterial activity observed against *A. baumannii* ATCC 17978 and *E. coli* ATCC 25922.

## 5 Conclusion

The results of this study provide new insights into the use of thiophene derivatives against Col-R *A. baumannii* and *E. coli*, where therapeutic options are limited. Despite the potential showed by this new family of antibacterial compounds, further studies are necessary to elucidate the *in vivo* mechanism of action of this chemical class and their implications for the host, as well as to determine an optimal dosage that achieves therapeutic efficacy in the treatment of severe infections caused by MDR-GNB.

## Data Availability

The data supporting the findings of this study are available from the corresponding author upon reasonable request.
