# Supplementary material for: Discovery of new antimicrobial thiophene derivatives with activity against drug-resistant Gram negative-bacteria
Source: Front Pharmacol. 2024 Aug 20;15:1412797. doi: 10.3389/fphar.2024.1412797 (PMC11368766; doi:10.3389/fphar.2024.1412797)
Supplement: Supplementary file 1 [file DataSheet1.PDF]

Table S1. Antibacterial activity of initial selection of compounds in *A. baumannii* and *E. coli* reference strains.

Compound	Chemical structure	MIC (mg/L)	
		A. baumannii **ATCC 17978**	***E. coli*** **ATCC 25922**
**SC380**	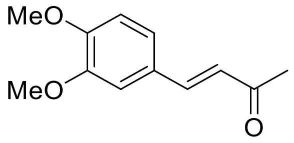	>64	>64
**SC628**	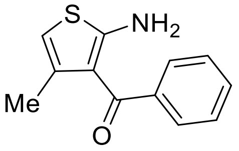	>64	>64
**MR1.44**	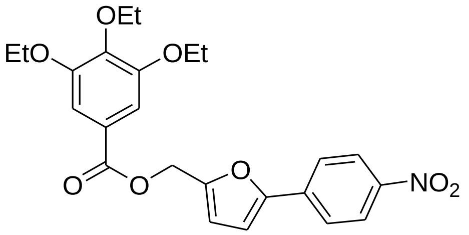	>64	>64
**MR1.51**	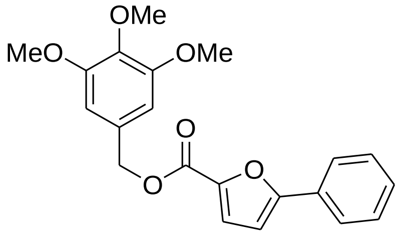	>64	>64
**MR2.21**	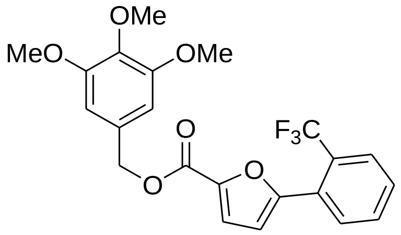	>64	>64
**MR2.39**	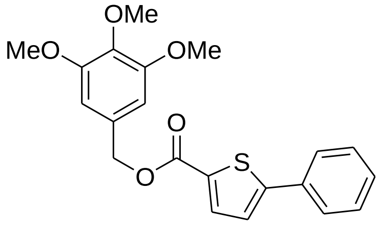	>64	>64
**MR2.50**	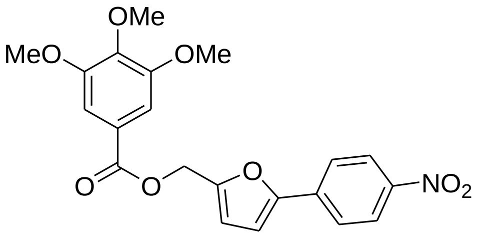	>64	>64
**VP3.21**	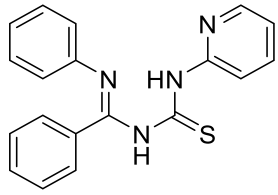	>64	>64

**MMT2.39**	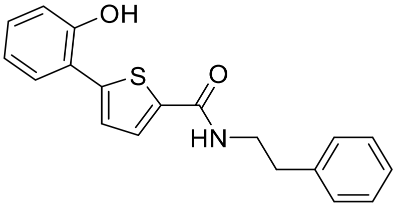	>64	>64
**AGR1.229**	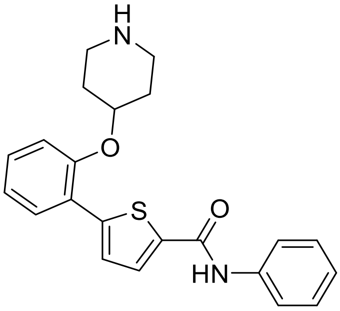	**32**	**64**
**AGR1.230**	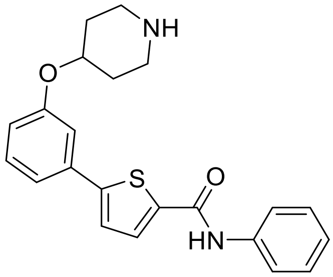	**16**	**16**
**AGR1.231**	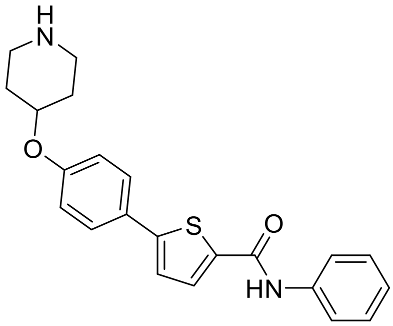	>64	>64

Table S2. Buser, Cosine, Kulczynski and Tanimoto similarity indexes for 12 compounds selected from similarity-based virtual screening of the MBC library. The two anticancer drugs, 4-hydroxytamoxifen (4OH-TAM) and raloxifene (RAL) were used as reference structures for similarity search.

**Comp.**	**BUSER**		**COSINE**		**KULCZYNSKI**		**TANIMOTO**		**MEAN***
	**4OH-TAM**	**RAL**	**4OH-TAM**	**RAL**	**4OH-TAM**	**RAL**	**4OH-TAM**	**RAL**	
SC380	0.89	0.67	0.39	0.16	0.44	0.26	0.21	0.05	0.49
SC628	0.82	0.81	0.20	0.38	0.20	0.47	0.11	0.18	0.46
VP3.21	0.83	0.67	0.24	0.13	0.24	0.16	0.13	0.06	0.37
MMT2.39	0.69	0.77	0.09	0.26	0.09	0.28	0.05	0.14	0.37
**AGR1.231**	**0.74**	**0.77**	**0.17**	**0.27**	**0.18**	**0.27**	**0.09**	**0.15**	**0.36**
MR2.50	0.72	0.77	0.13	0.27	0.14	0.28	0.07	0.15	0.36
MR1.51	0.72	0.77	0.12	0.26	0.12	0.28	0.06	0.14	0.36
MR1.44	0.74	0.77	0.16	0.26	0.17	0.27	0.08	0.14	0.36
**AGR1.230**	**0.73**	**0.76**	**0.16**	**0.26**	**0.17**	**0.26**	**0.08**	**0.14**	**0.36**
MR2.21	0.71	0.76	0.12	0.25	0.12	0.26	0.06	0.14	0.35
**AGR1.229**	**0.72**	**0.76**	**0.16**	**0.25**	**0.17**	**0.25**	**0.08**	**0.14**	**0.35**
MR2.39	0.72	0.77	0.12	0.26	0.12	0.28	0.06	0.14	0.23

* For each compound, mean values were calculated considering the higher similarity value from each metric.

Table S3. Docking score of thiophene derivative **4** binding on porins of *A. baumannii* and *E. coli*.

	Porins	Docking score	Binding amino acids
A. baumannii	OmpA	-3.691	Asp90, Gln45
	Omp33	-4.023	Glu189, Glu43
	OmpW	-2.732	Asp 149, Trp 153
	CarO1	-4.772	Tyr108
E. coli	OmpA	-4.755	Lys12, Asp56
	OmpC	-5.556	Arg74, Tyr94, Phe110, Asp105
	OmpW	-8.161	Phe43
	OmpF	-4.944	Asp113, Gly110

Figure S1. HPLC chromatograms and MS of tested compounds

SC380 *((E)-4-(3,4-dimethoxyphenyl)but-3-en-2-one))*

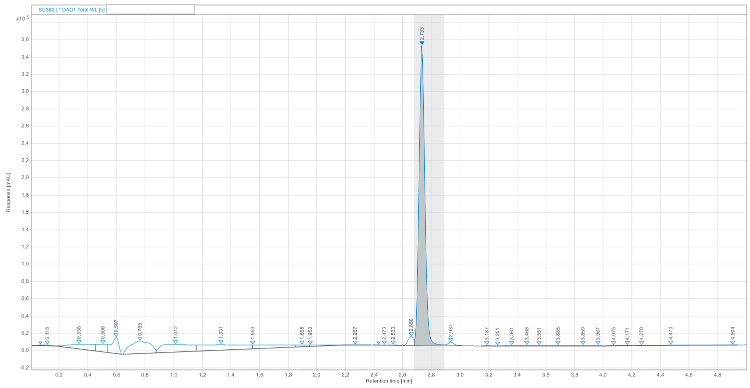

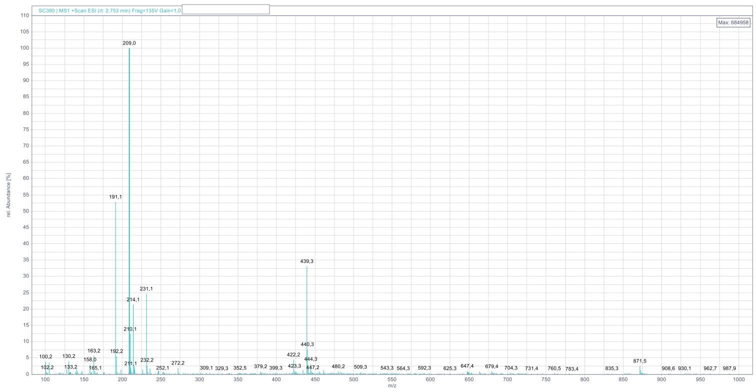

SC628 *((2-amino-4-methylthiophen-3-yl)(phenyl)methanone)*

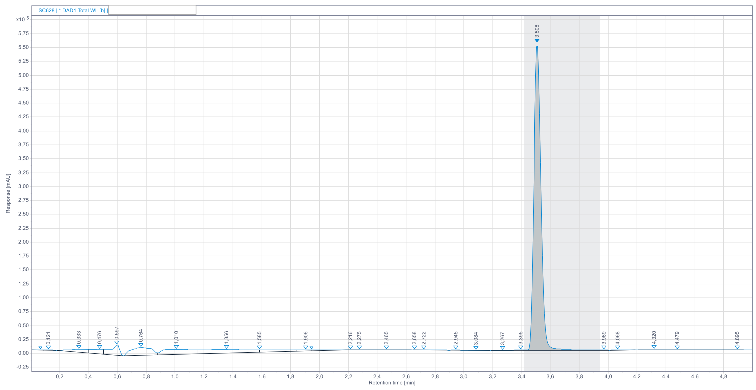

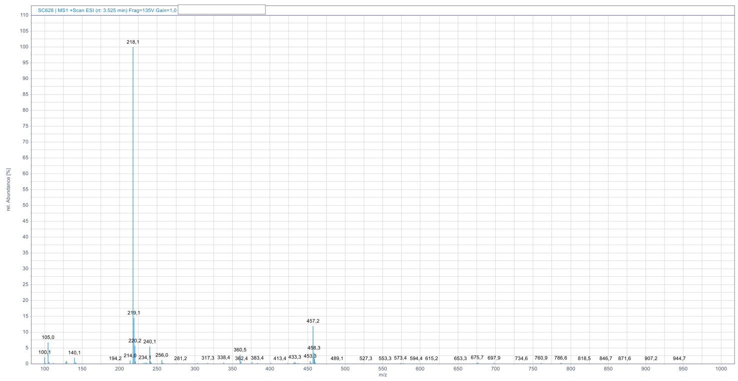

MR1.44 *((5-(4-nitrophenyl)furan-2-yl)methyl 3,4,5-triethoxybenzoate)*

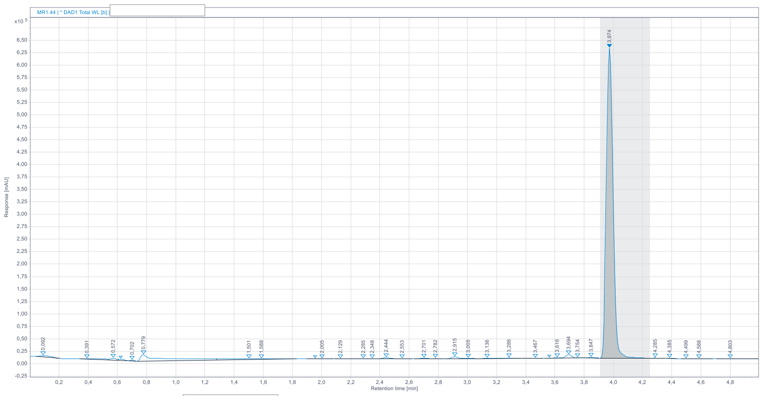

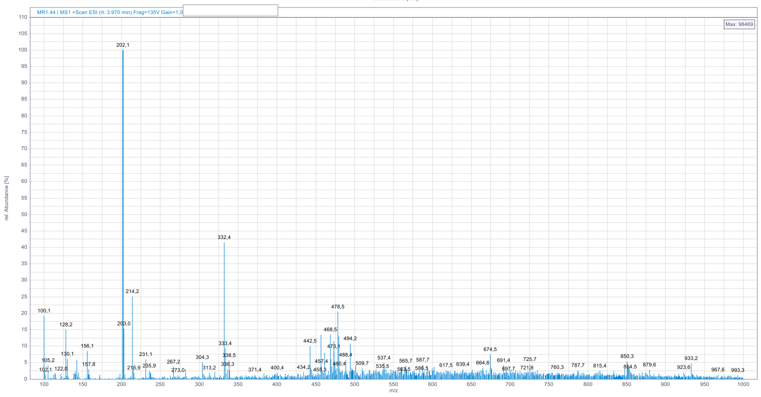

MR1.51 *(3,4,5-trimethoxybenzyl 5-phenylfuran-2-carboxylate)*

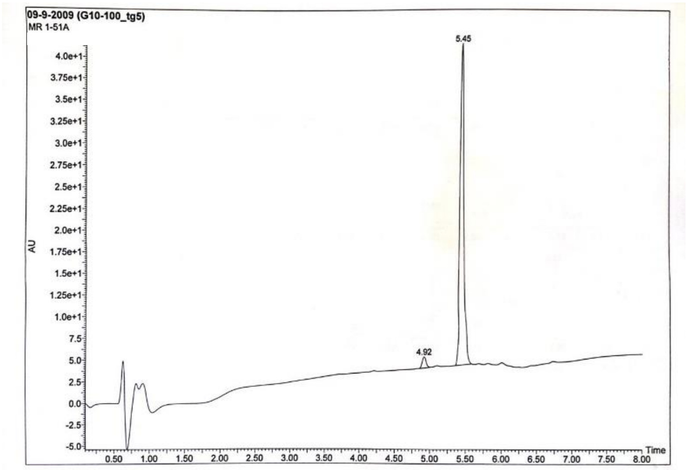

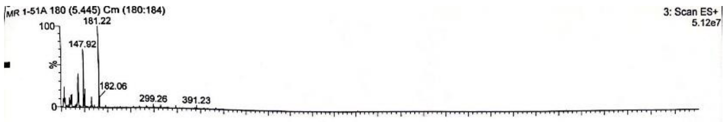

MR2.21 *(3,4,5-trimethoxybenzyl 5-(2-(trifluoromethyl)phenyl)furan-2-carboxylate)*

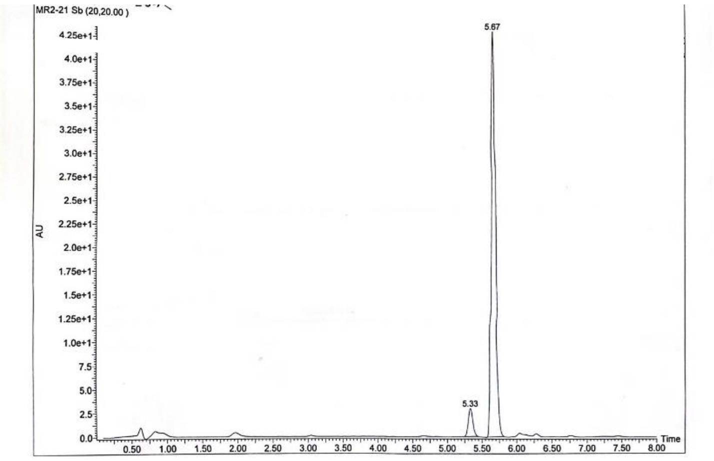

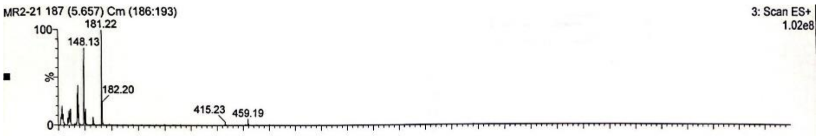

MR2.39 *(3,4,5-trimethoxybenzyl 5-phenylthiophene-2-carboxylate)*

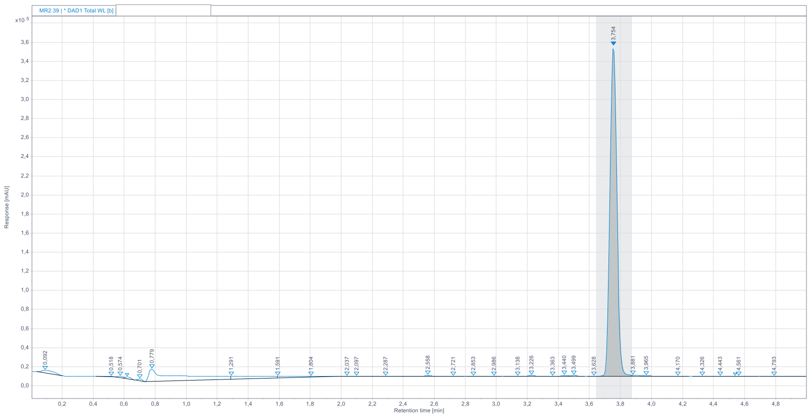

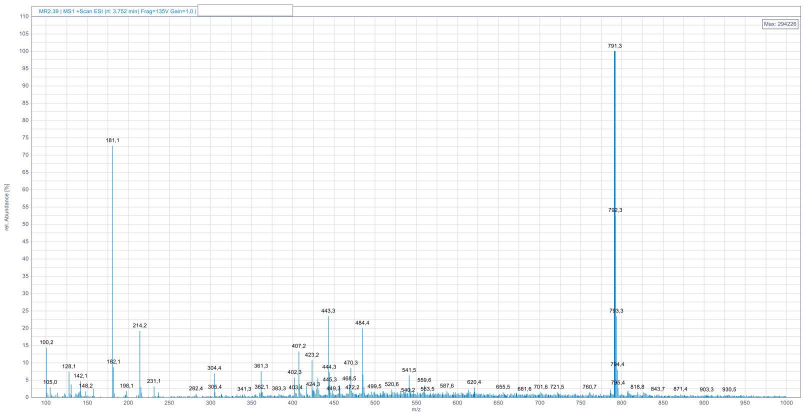

MR2.50 *((5-(4-nitrophenyl)furan-2-yl)methyl 3,4,5-trimethoxybenzoate)*

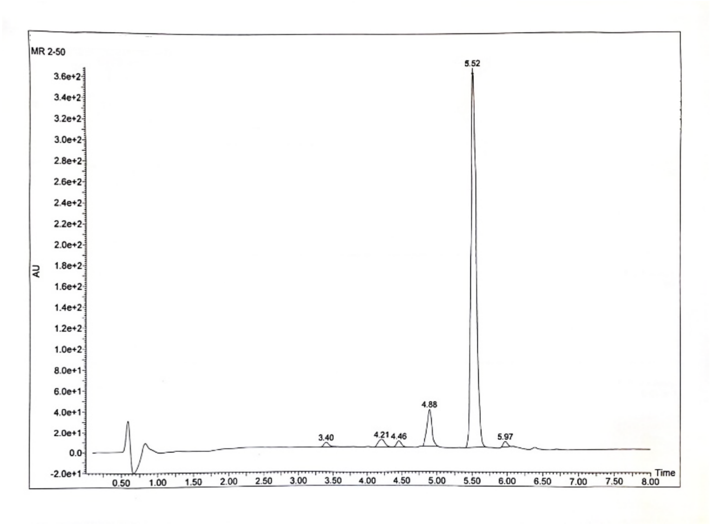

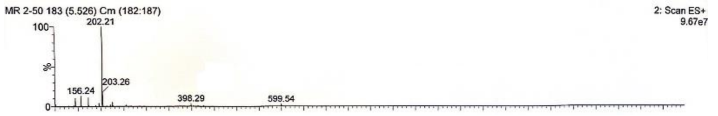

VP3.21 *((E)-N'-phenyl-N-(pyridin-2-ylcarbamothioyl)benzimidamide)*

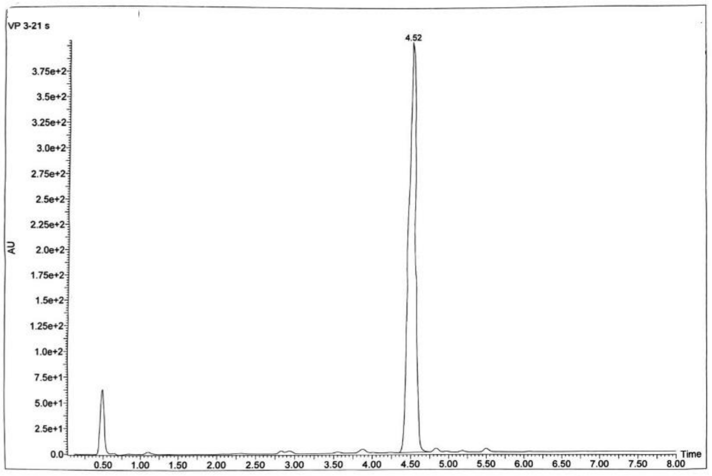

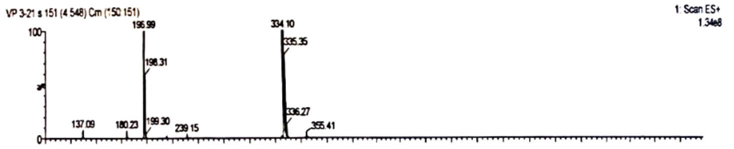

MMT2.39 *(5-(2-Hydroxyphenyl)-N-phenethylthiophene-2-carboxamide)*

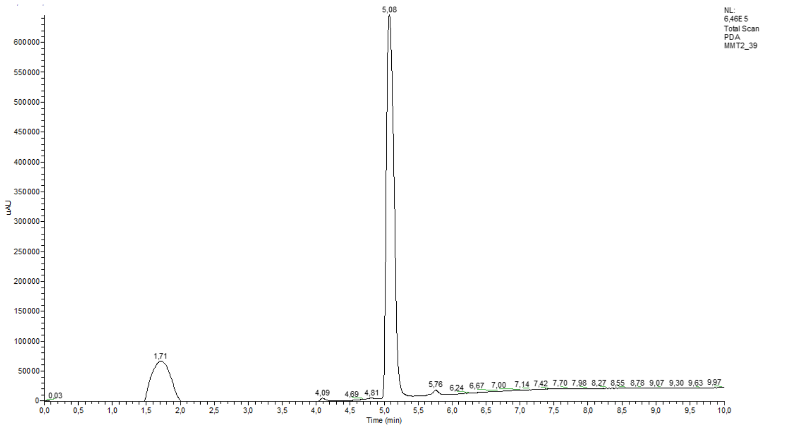

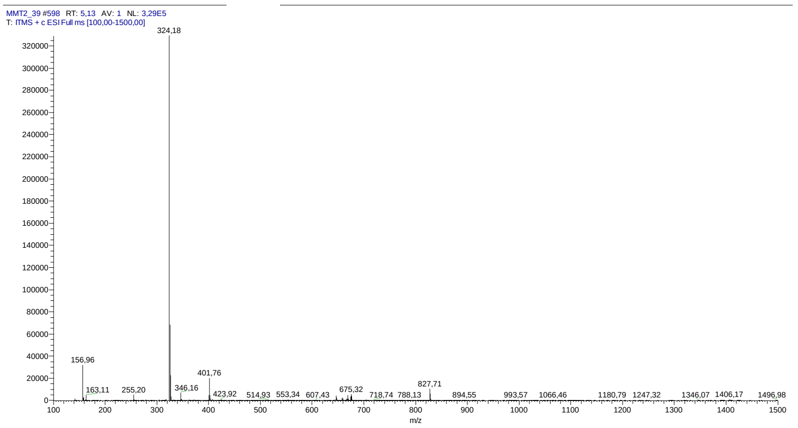

AGR1.229 (1) *(N-Phenyl-5-(2-(piperidin-4-yloxy)phenyl)thiophene-2-carboxamide)*

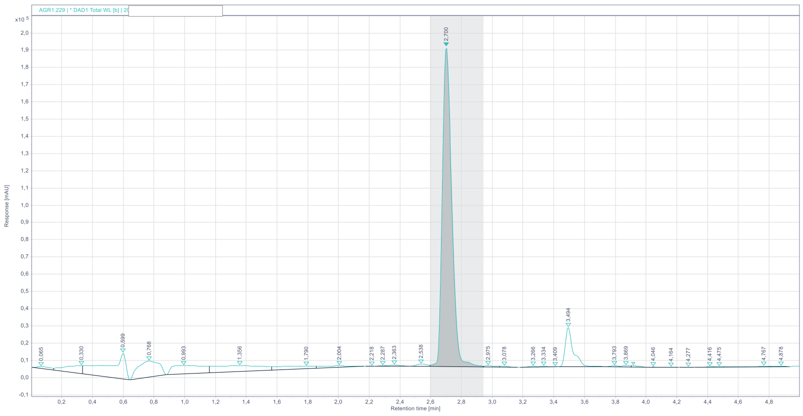

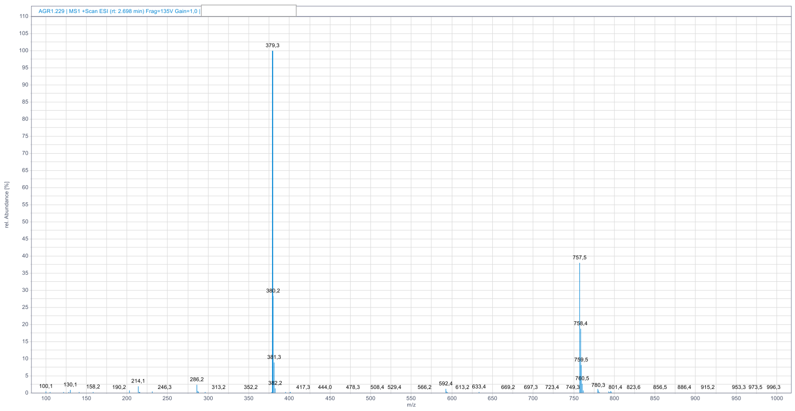

AGR1.230 (2) *(N-Phenyl-5-(3-(piperidin-4-yloxy)phenyl)thiophene-2-carboxamide)*

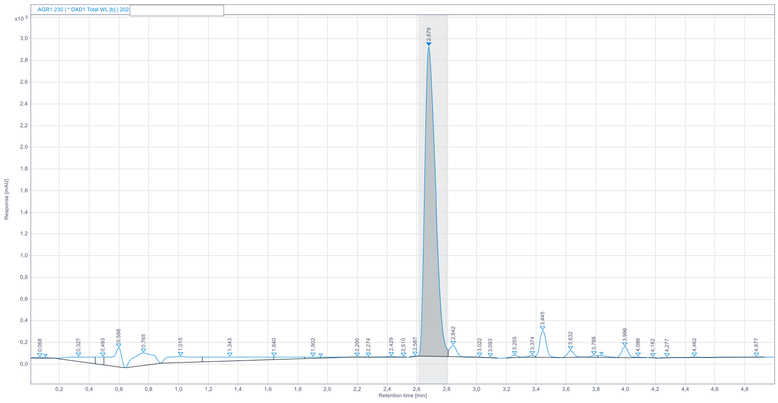

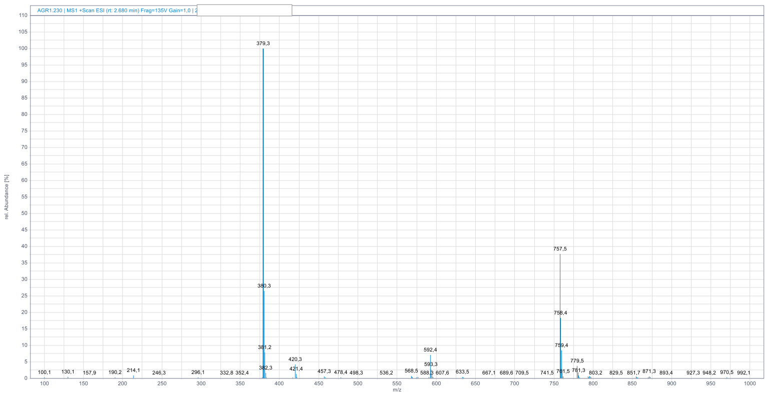

AGR1.231 (3) *(N-Phenyl-5-(4-(piperidin-4-yloxy)phenyl)thiophene-2-carboxamide)*

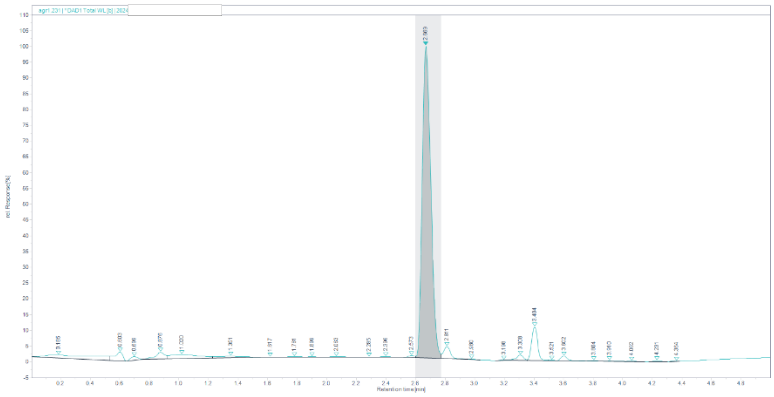

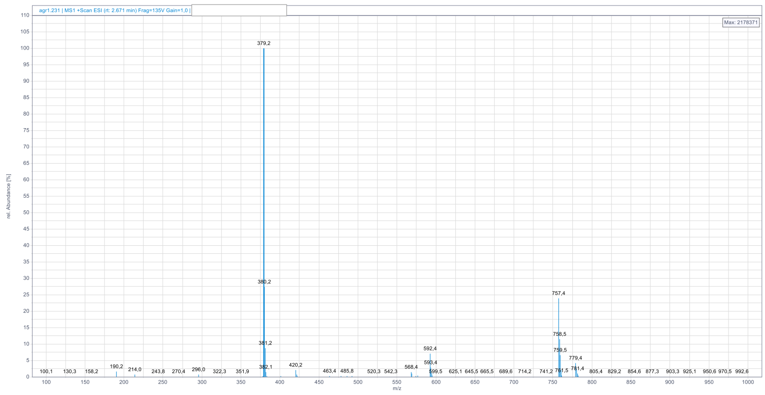

4 *(N-(4-Chlorophenyl)-5-(2-(piperidin-4-yloxy)phenyl)thiophene-2-carboxamide)*

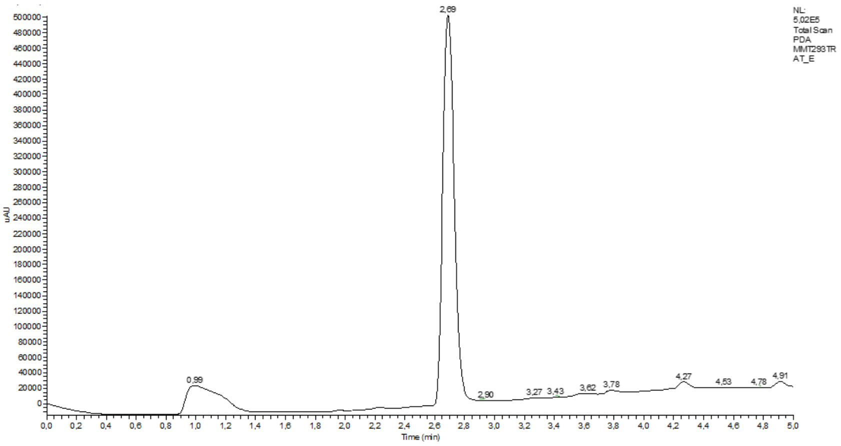

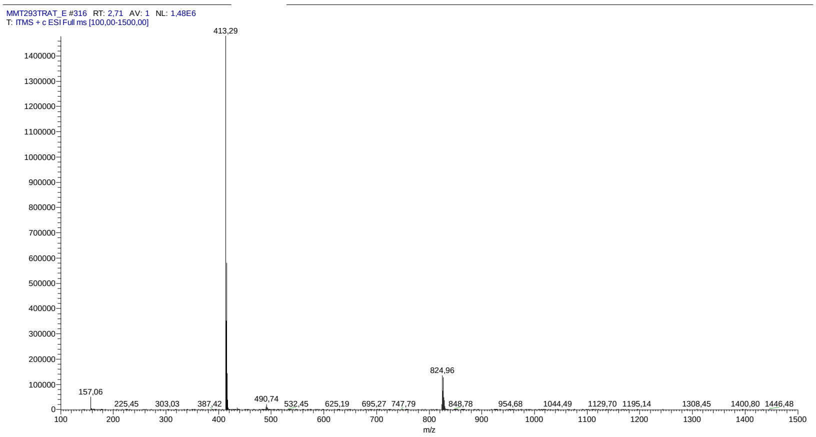

5 *(N-(4-Chlorophenyl)-5-(3-(piperidin-4-yloxy)phenyl)thiophene-2-carboxamide)*

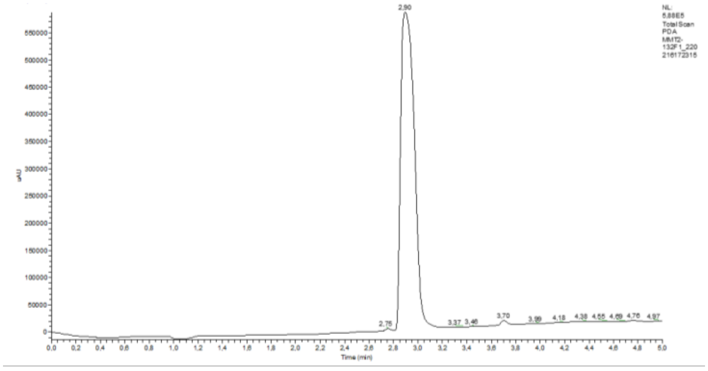

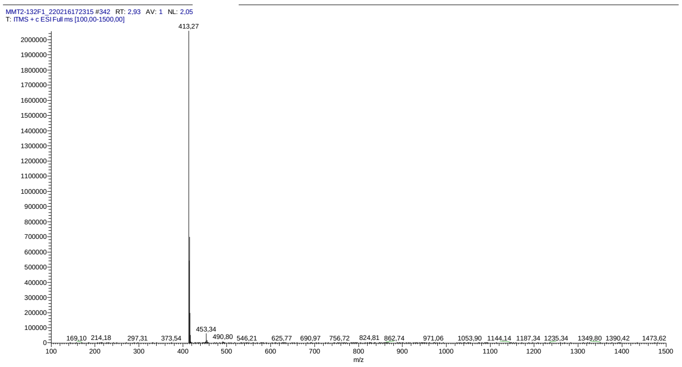

6 *(N-(4-Chlorophenyl)-5-(4-(piperidin-4-yloxy)phenyl)thiophene-2-carboxamide)*

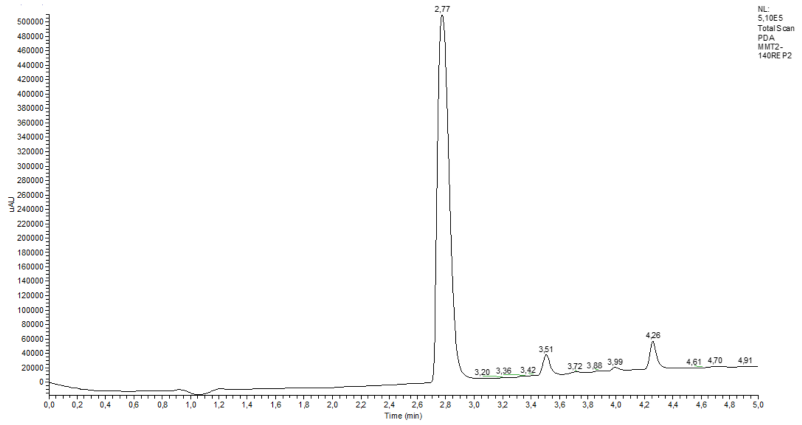

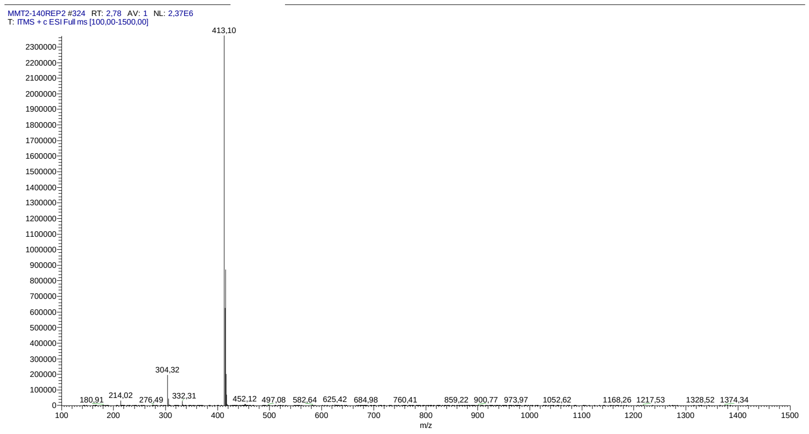

7 *(N-(3-Chlorophenyl)-5-(2-(piperidin-4-yloxy)phenyl)thiophene-2-carboxamide)*

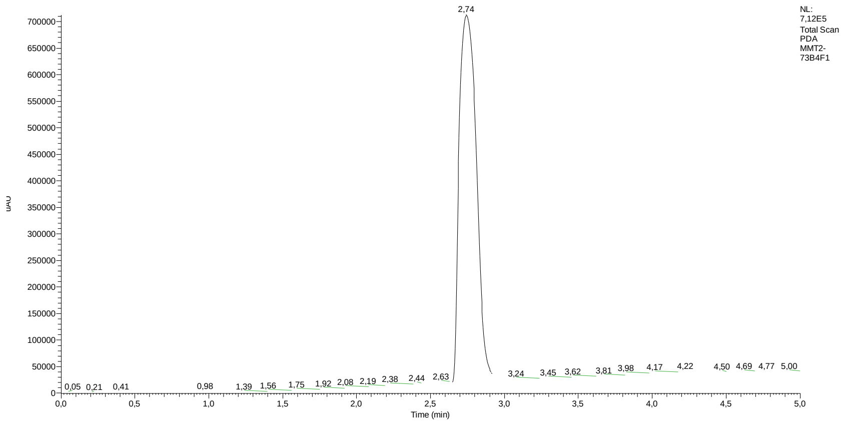

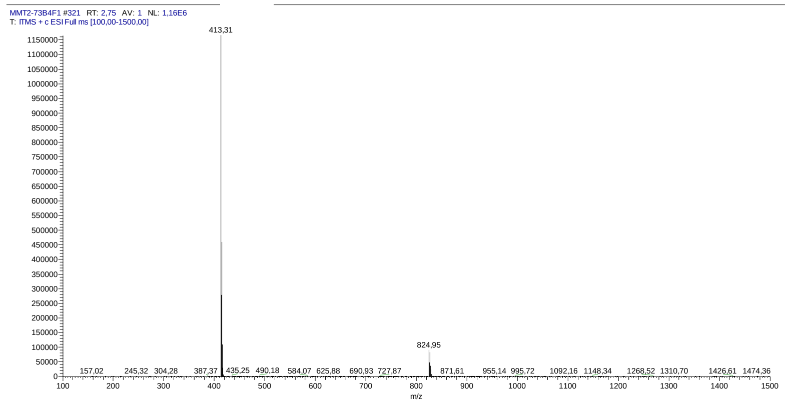

8 *(N-(3-Chlorophenyl)-5-(3-(piperidin-4-yloxy)phenyl)thiophene-2-carboxamide)*

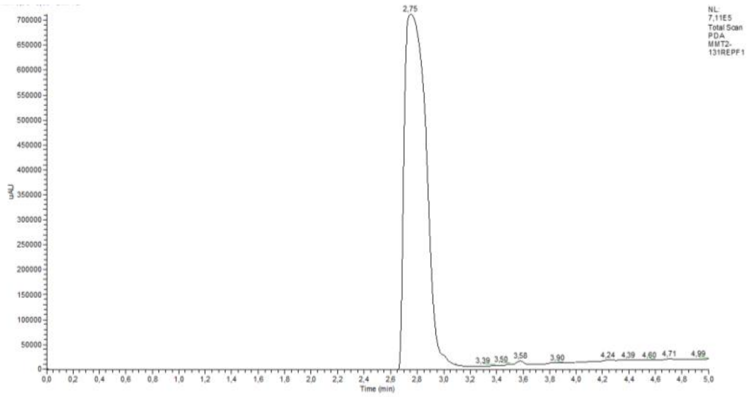

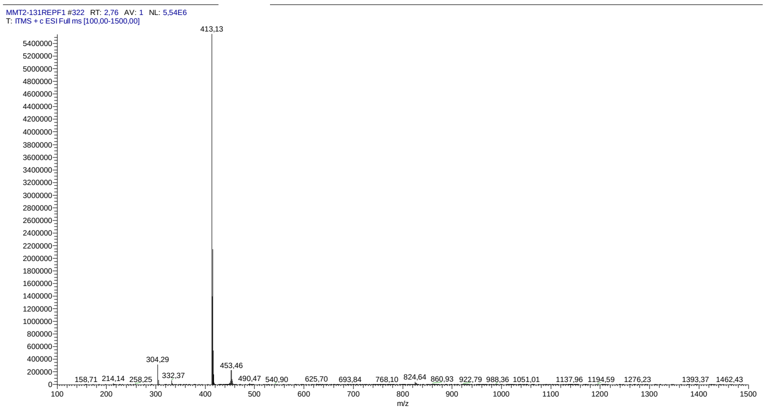

9 *(N-(3-Chlorophenyl)-5-(4-(piperidin-4-yloxy)phenyl)thiophene-2-carboxamide)*

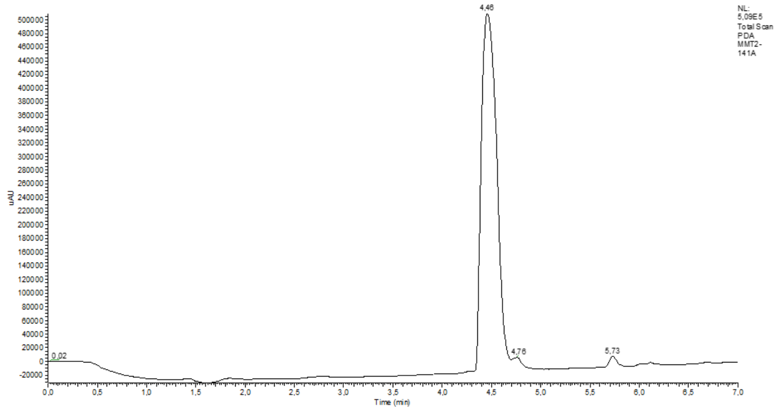

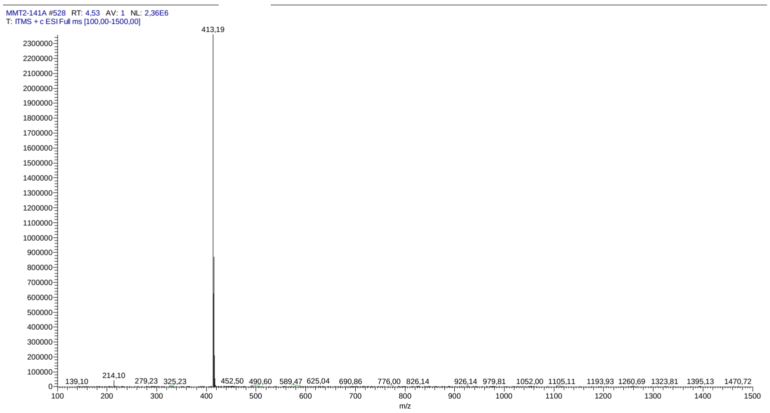

10 *(N-Phenethyl-5-(2-(piperidin-4-yloxy)phenyl)thiophene-2-carboxamide)*

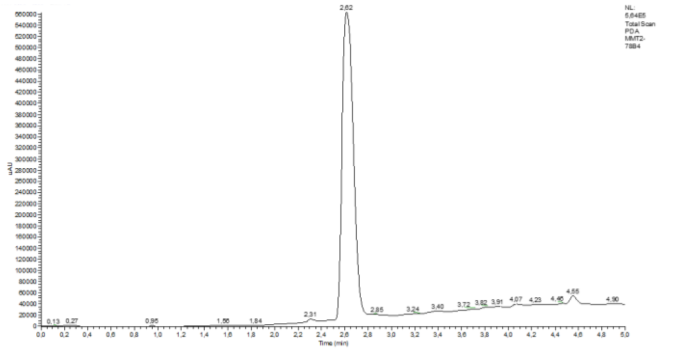

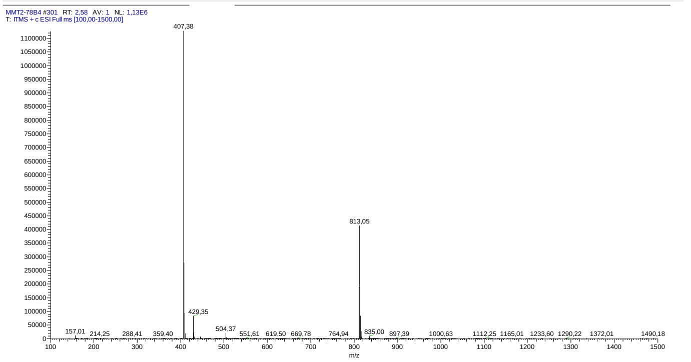

11 *(N-Phenethyl-5-(3-(piperidin-4-yloxy)phenyl)thiophene-2-carboxamide)*

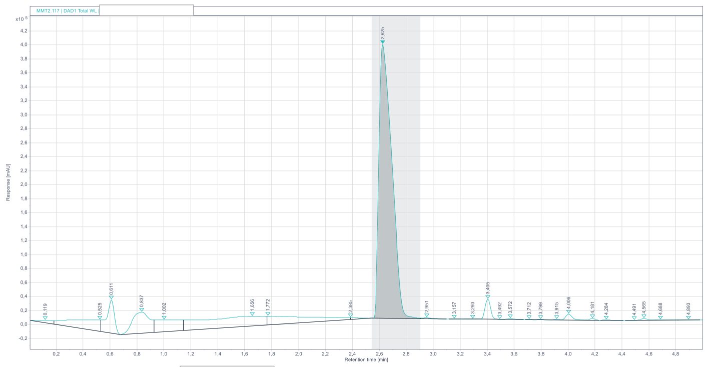

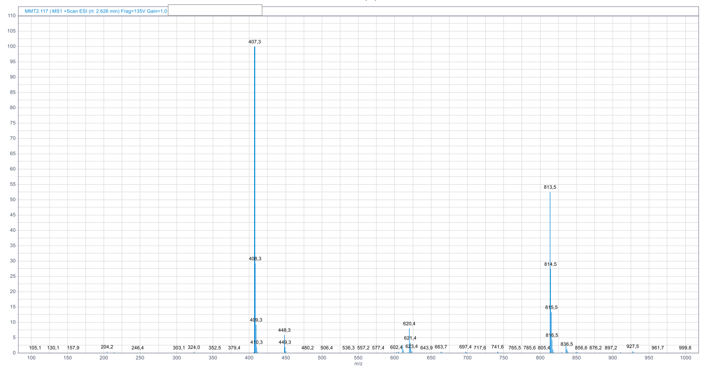

12 *(N-Phenethyl-5-(4-(piperidin-4-yloxy)phenyl)thiophene-2-carboxamide)*

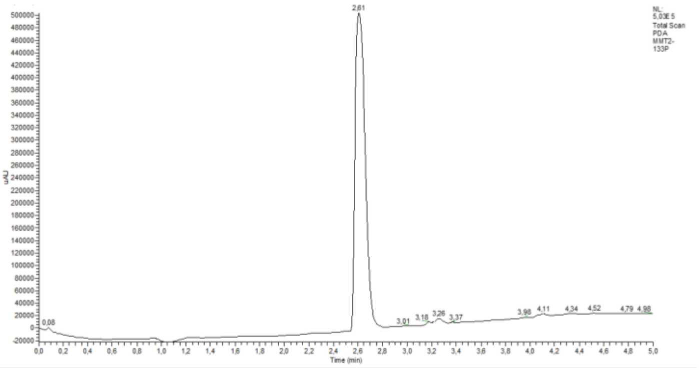

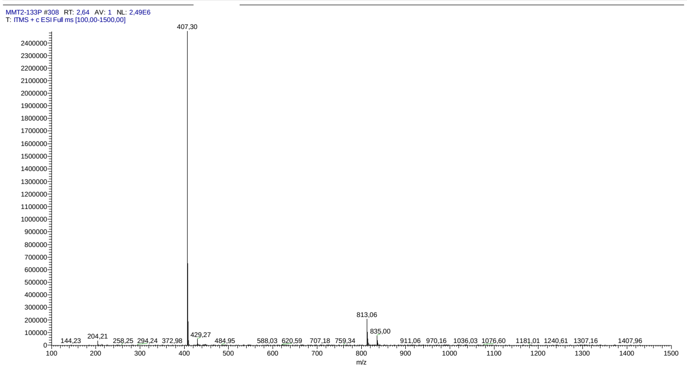

13 *(N-(2-(1-Benzylpiperidin-4-yl)ethyl)-5-(2-(piperidin-4-yloxy)phenyl)thiophene-2-carboxamide)*

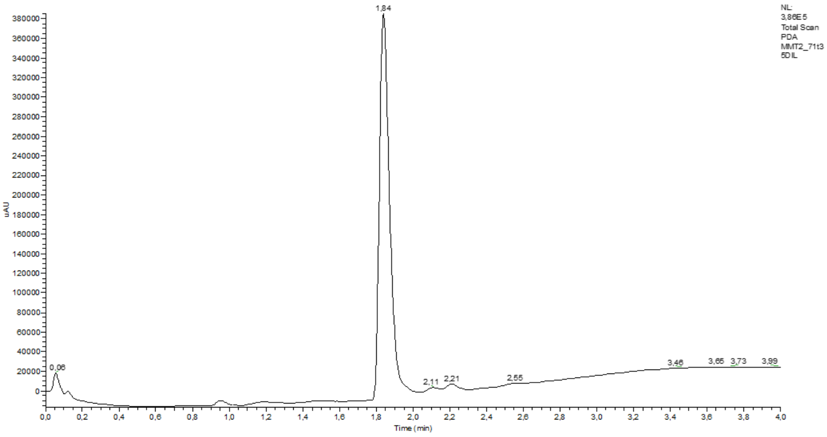

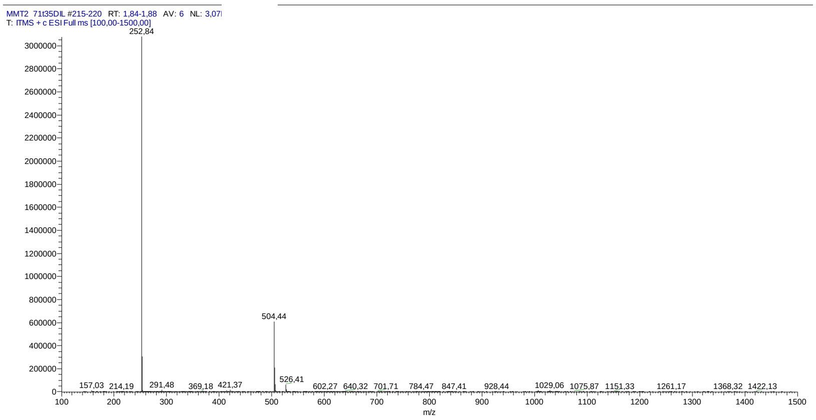

14 *(N-(2-(1-Benzylpiperidin-4-yl)ethyl)-5-(4-(piperidin-4-yloxy)phenyl)thiophene-2-carboxamide)*

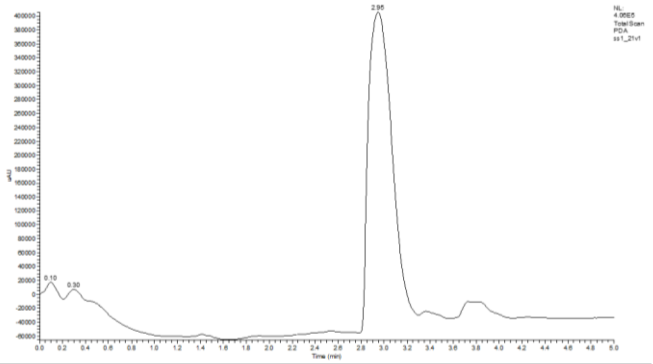

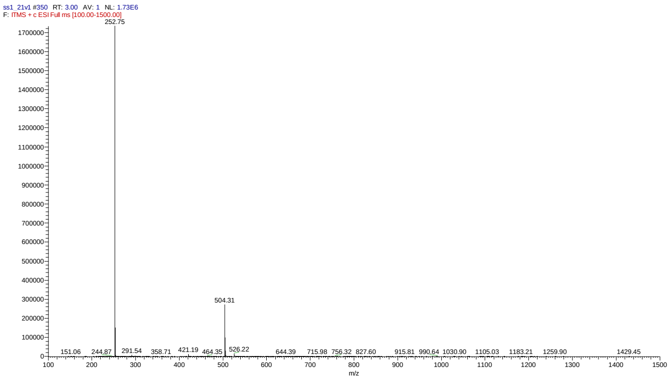

15 *(N-(4-Morpholinophenyl)-5-(2-(piperidin-4-yloxy)phenyl)thiophene-2-carboxamide)*

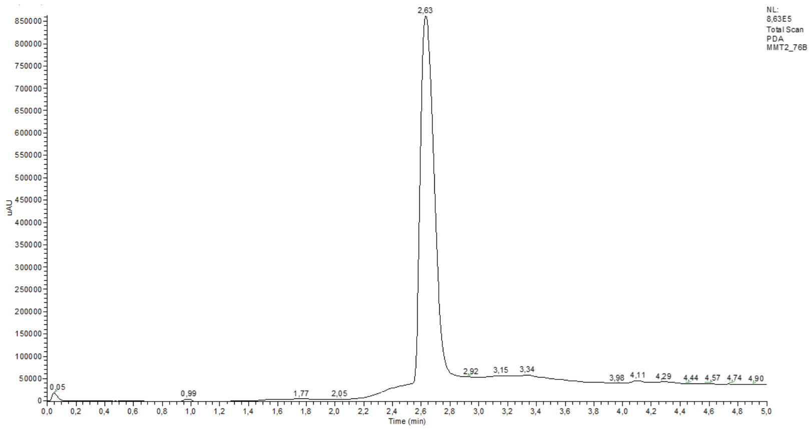

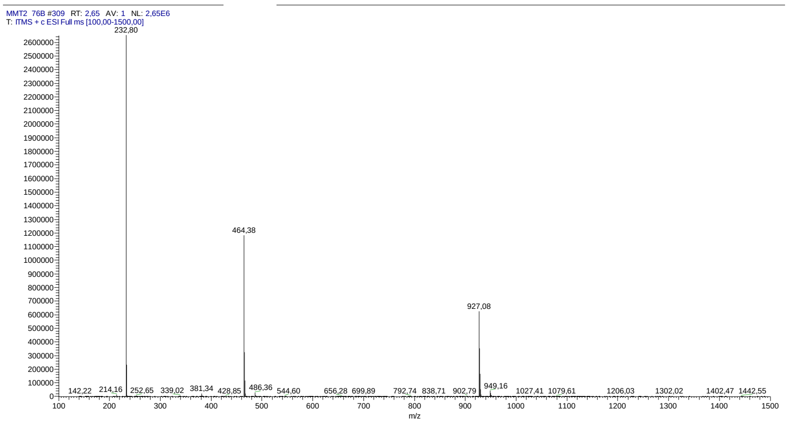

16 *(N-Phenyl-5-(2-(piperidin-4-yloxy)phenyl)furan-2-carboxamide)*

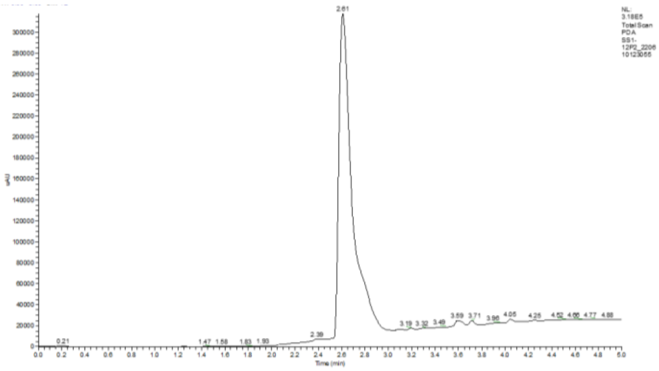

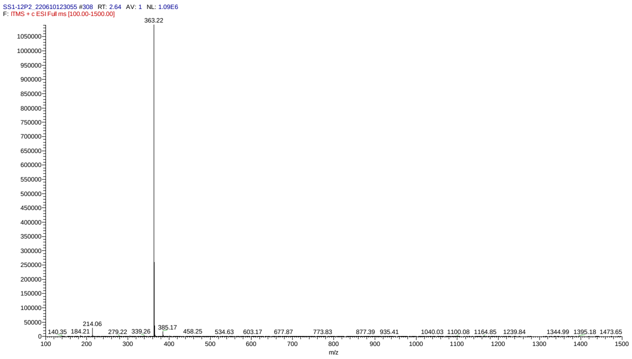

17 *(N-Phenyl-5-(3-(piperidin-4-yloxy)phenyl)furan-2-carboxamide)*

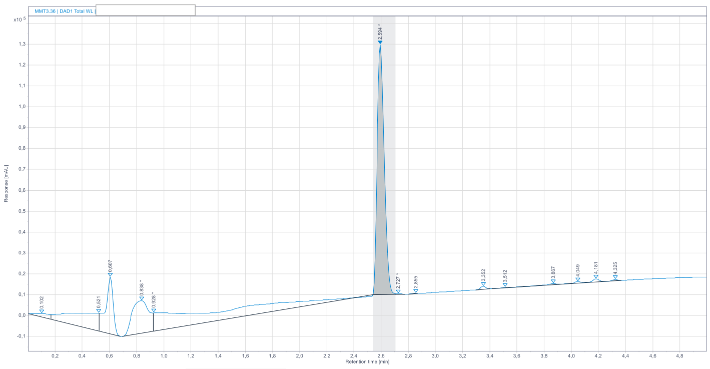

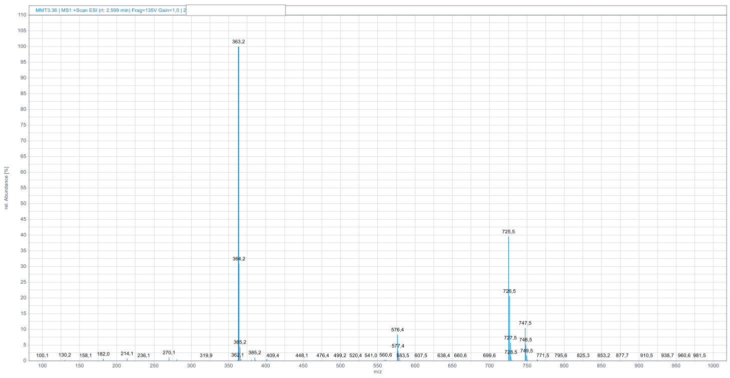

18 *(N-Phenyl-5-(4-(piperidin-4-yloxy)phenyl)furan-2-carboxamide)*

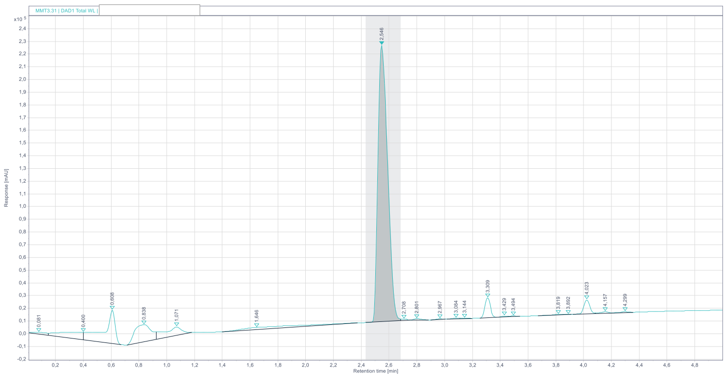

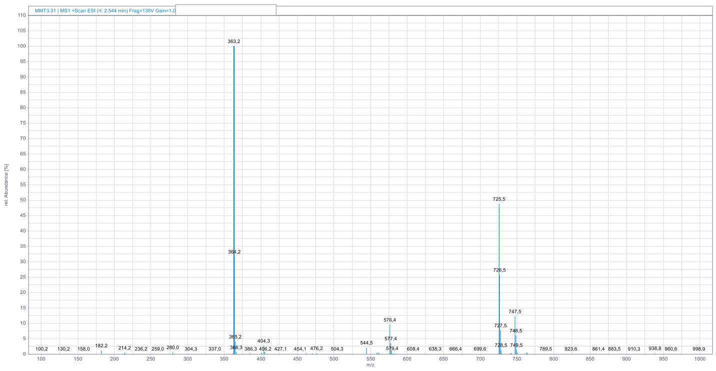

19 *(N-(4-Chlorophenyl)-5-(2-(piperidin-4-yloxy)phenyl)furan-2-carboxamide)*

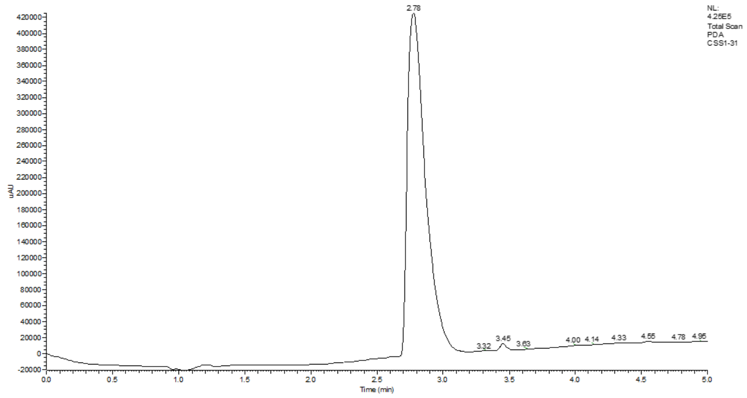

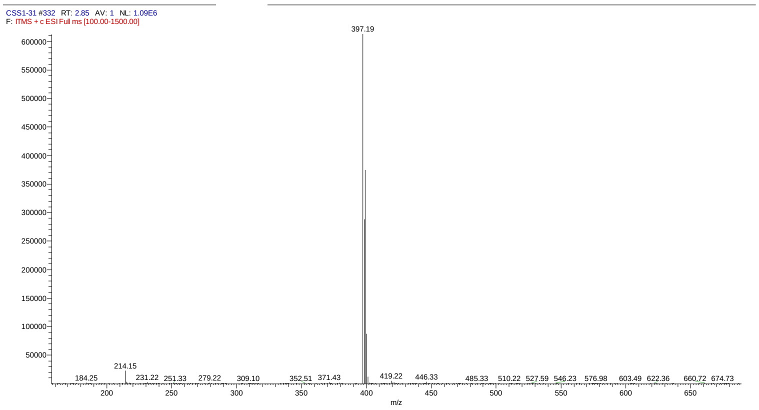

20 *(N-(3-Chlorophenyl)-5-(4-(piperidin-4-yloxy)phenyl)furan-2-carboxamide)*

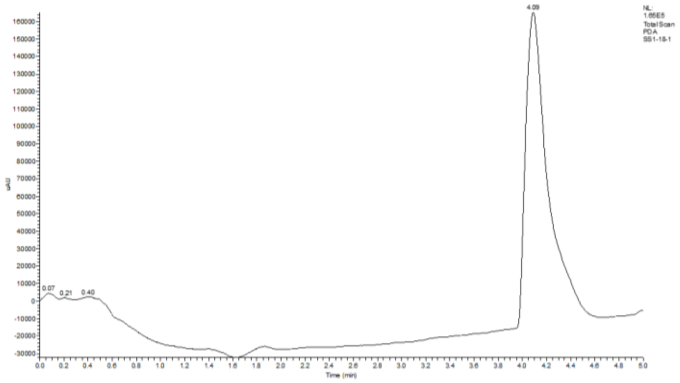

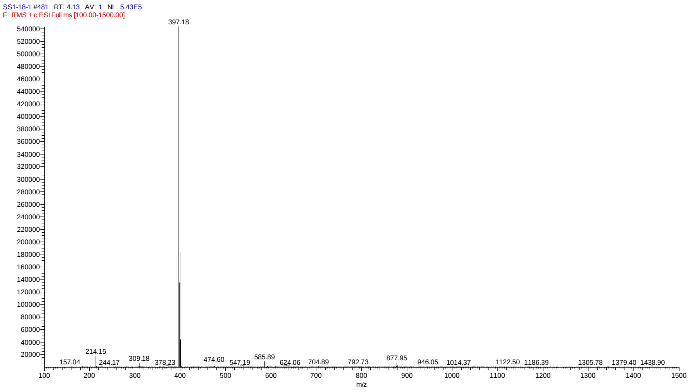

21 *(N-Phenethyl-5-(2-(piperidin-4-yloxy)phenyl)furan-2-carboxamide)*

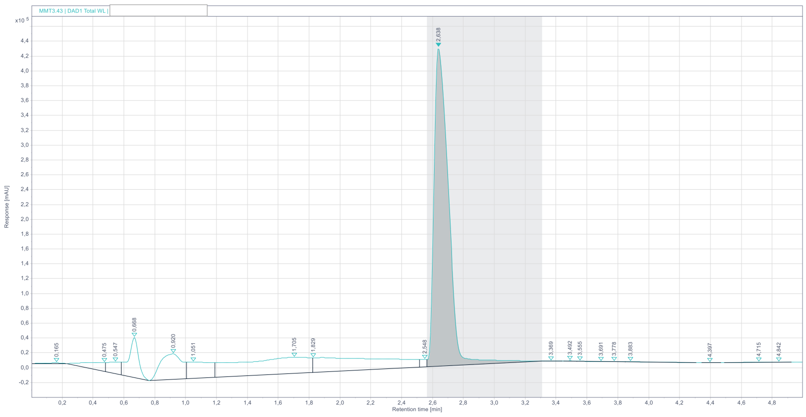

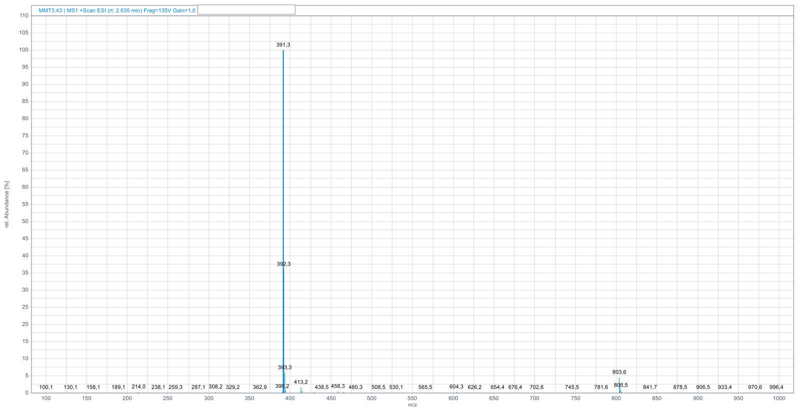

22 *(N-(2-(1-Benzylpiperidin-4-yl)ethyl)-5-(2-(piperidin-4-yloxy)phenyl)furan-2-carboxamide)*

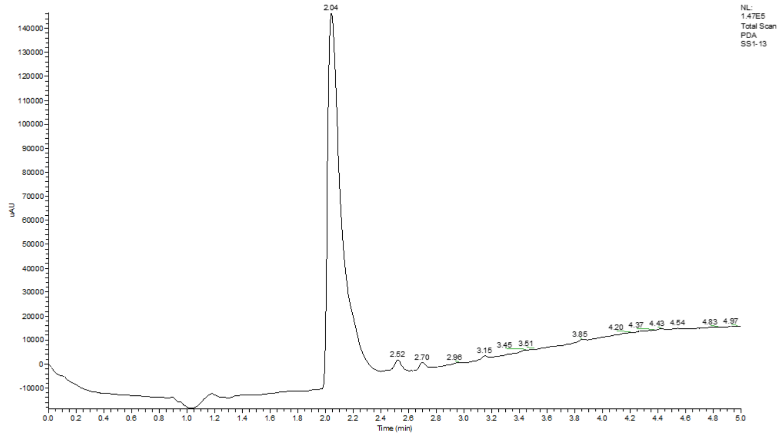

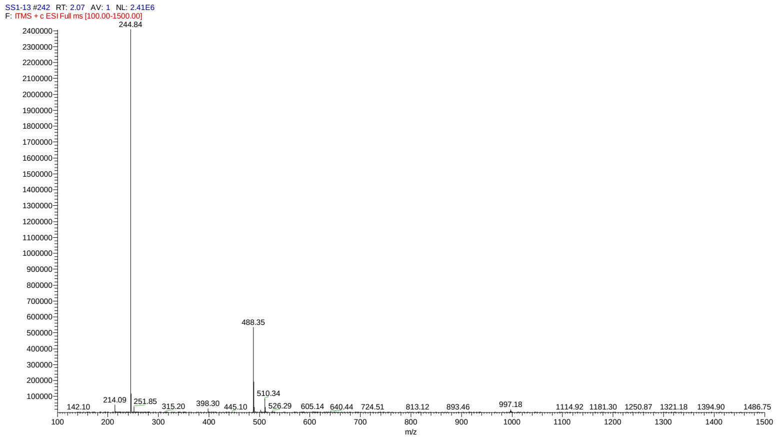

23 *(N-(2-(1-Benzylpiperidin-4-yl)ethyl)-5-(4-(piperidin-4-yloxy)phenyl)furan-2-carboxamide)*

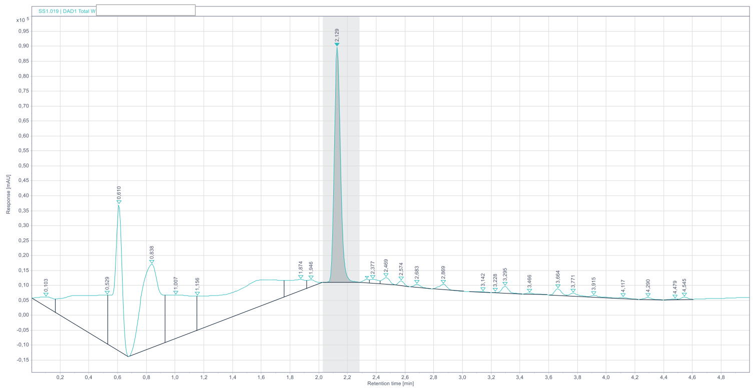

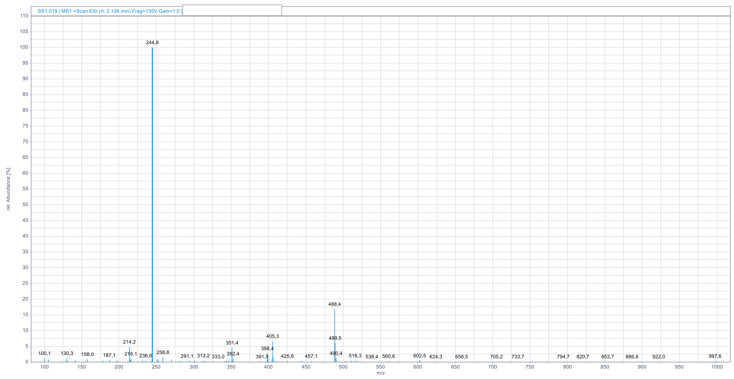

24 (*N-Phenyl-2-(3-(piperidin-4-yloxy)phenyl)thiazole-5-carboxamide)*

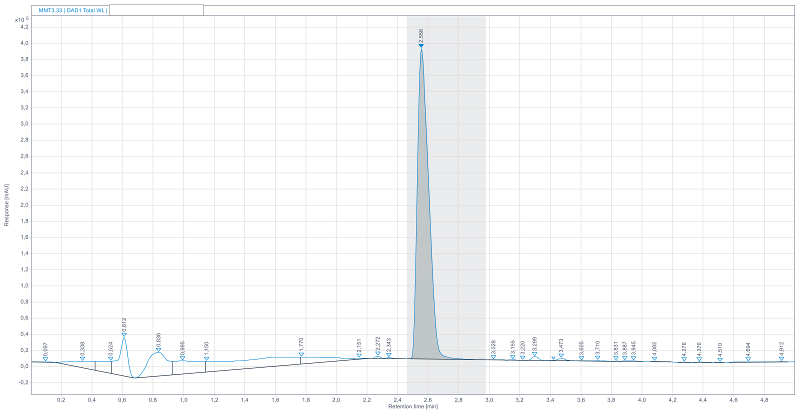

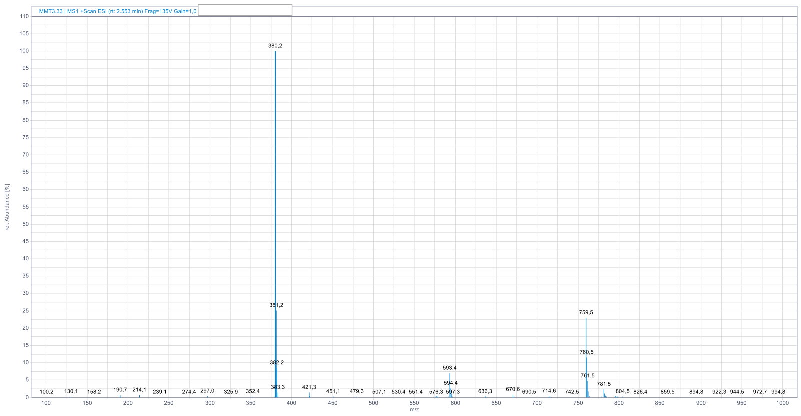

25 *(N-Phenyl-2-(4-(piperidin-4-yloxy)phenyl)thiazole-5-carboxamide)*

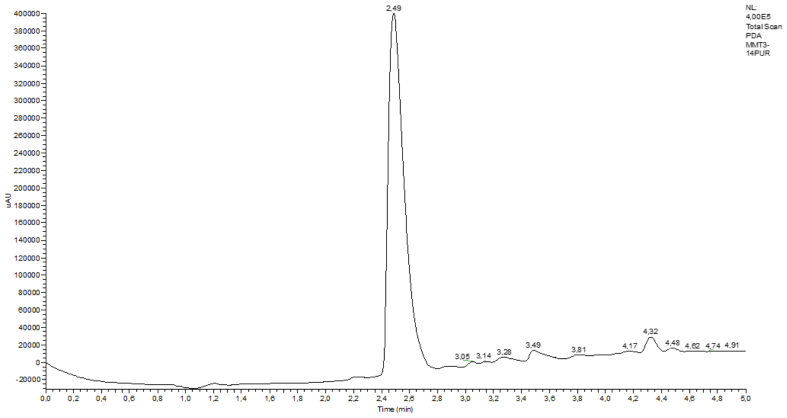

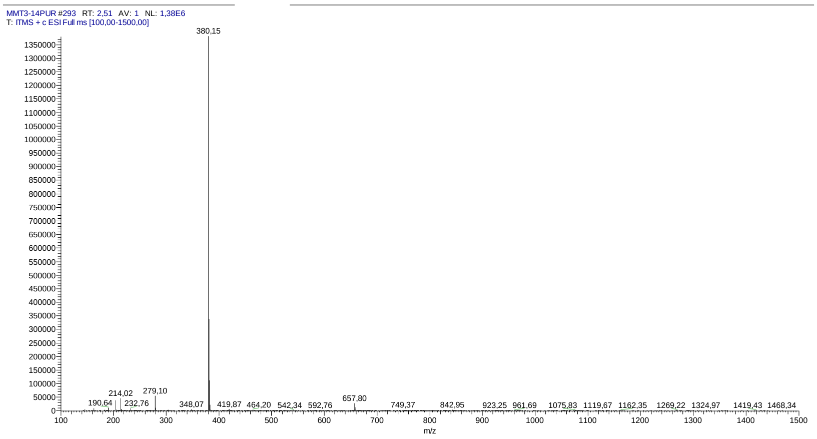

26 *(N-Phenethyl-2-(2-(piperidin-4-yloxy)phenyl)thiazole-5-carboxamide)*

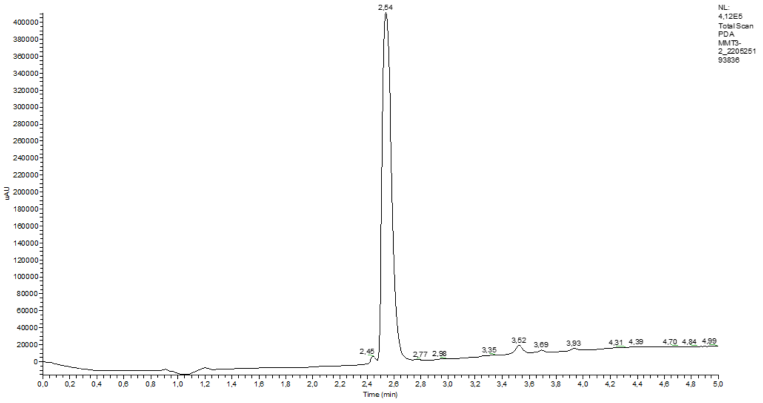

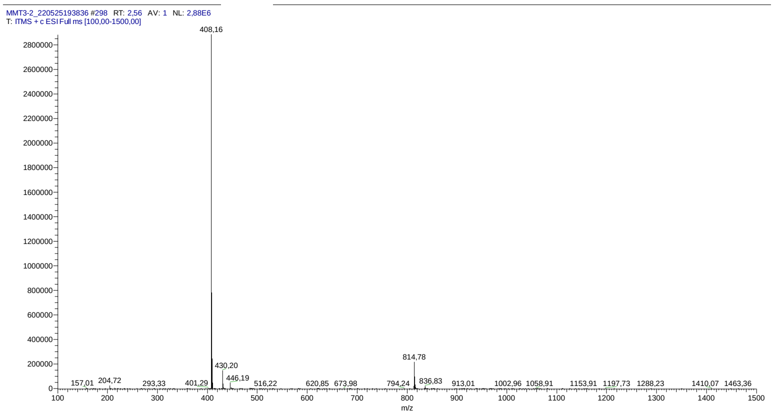

27 *(N-(2-(1-Benzylpiperidin-4-yl)ethyl)-2-(4-(piperidin-4-yloxy)phenyl)thiazole-5-carboxamide)*

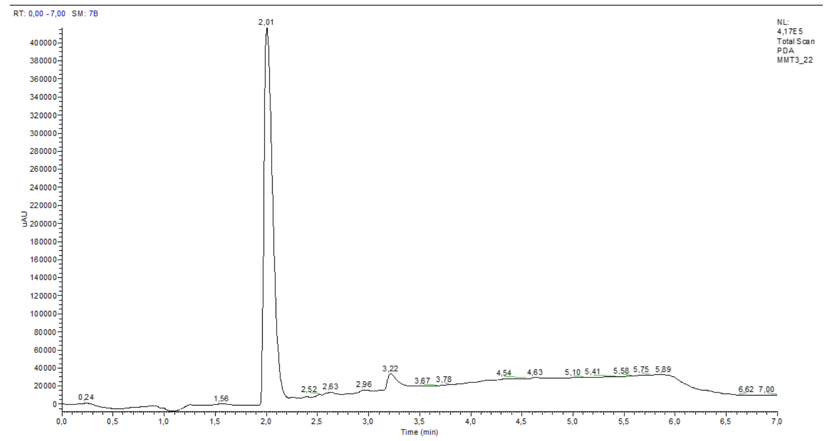

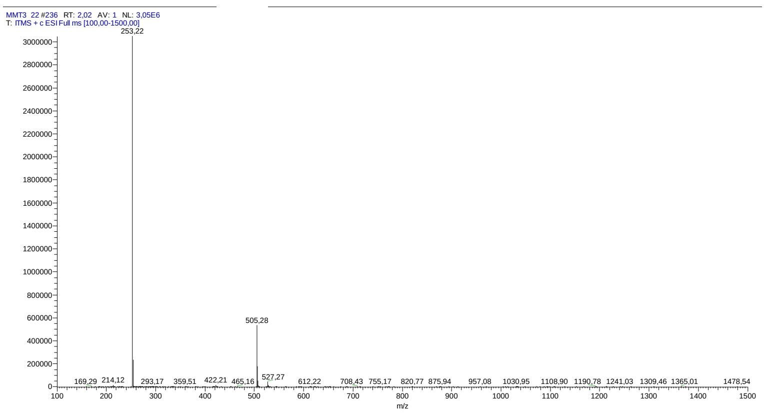

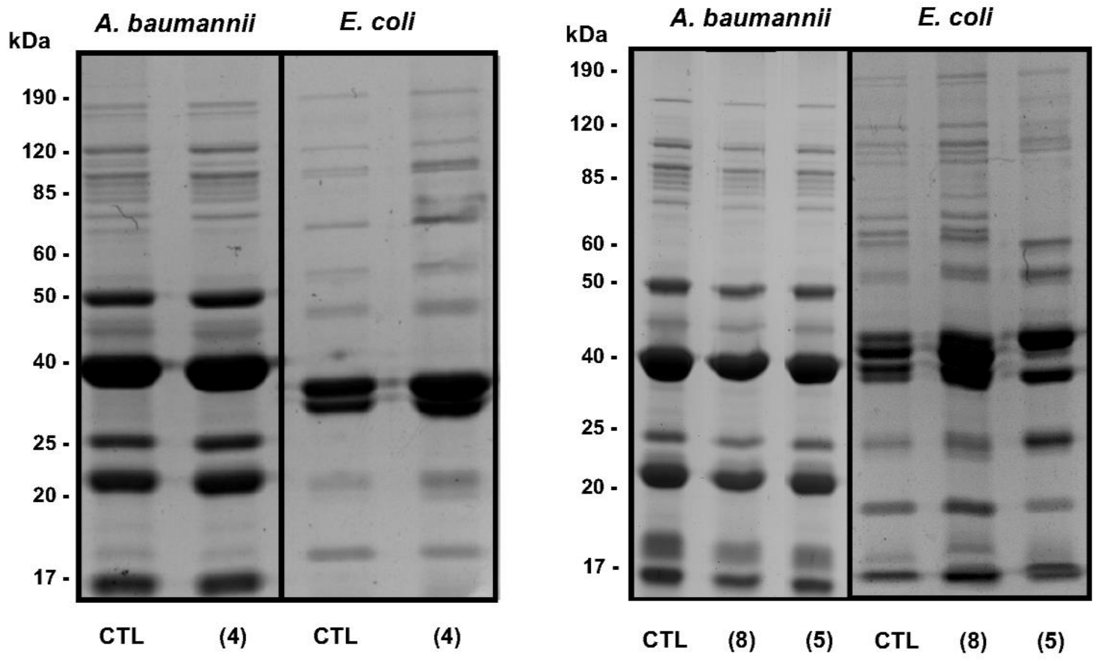

Figure S2. Outer membrane profile of clinical colistin-resistant *A. baumannii* and *E. coli* after treated with thiophene derivatives 4, 5 and 8. SDS-PAGE of the outer membrane proteins of *A. baumannii* and *E. coli* without or with thiophenes **4**, **5** and **8** treatment. CTL: control. **A. baumannii* Ab21 and *E. coli* MCR1^{+} strain were treated with thiophene **4**. ***A. baumannii* Ab11 and *E. coli* R6 MCR1 strains were treated with thiophenes **5** and **8**.
